# Advancing animal tuberculosis surveillance using culture-independent long-read whole-genome sequencing

**DOI:** 10.3389/fmicb.2023.1307440

**Published:** 2023-11-21

**Authors:** Giovanni Ghielmetti, Johannes Loubser, Tanya J. Kerr, Tod Stuber, Tyler Thacker, Lauren C. Martin, Michaela A. O'Hare, Sinegugu K. Mhlophe, Abisola Okunola, Andre G. Loxton, Robin M. Warren, Mark H. Moseley, Michele A. Miller, Wynand J. Goosen

**Affiliations:** ^1^Division of Molecular Biology and Human Genetics, South African Medical Research Council Centre for Tuberculosis Research, Faculty of Medicine and Health Sciences, Stellenbosch University, Cape Town, South Africa; ^2^Section of Veterinary Bacteriology, Institute for Food Safety and Hygiene, Vetsuisse Faculty, University of Zurich, Zurich, Switzerland; ^3^National Veterinary Services Laboratories, Veterinary Services, Animal and Plant Health Inspection Service, United States Department of Agriculture, Ames, IA, United States; ^4^School of Biological Sciences, University of Aberdeen, Aberdeen, United Kingdom

**Keywords:** adaptive sampling, African buffaloes, culture-independent, *Mycobacterium bovis*, next-generation sequencing, whole-genome sequencing

## Abstract

Animal tuberculosis is a significant infectious disease affecting both livestock and wildlife populations worldwide. Effective disease surveillance and characterization of *Mycobacterium bovis (M. bovis)* strains are essential for understanding transmission dynamics and implementing control measures. Currently, sequencing of genomic information has relied on culture-based methods, which are time-consuming, resource-demanding, and concerning in terms of biosafety. This study explores the use of culture-independent long-read whole-genome sequencing (WGS) for a better understanding of *M. bovis* epidemiology in African buffaloes (*Syncerus caffer*). By comparing two sequencing approaches, we evaluated the efficacy of Illumina WGS performed on culture extracts and culture-independent Oxford Nanopore adaptive sampling (NAS). Our objective was to assess the potential of NAS to detect genomic variants without sample culture. In addition, culture-independent amplicon sequencing, targeting mycobacterial-specific housekeeping and full-length 16S rRNA genes, was applied to investigate the presence of microorganisms, including nontuberculous mycobacteria. The sequencing quality obtained from DNA extracted directly from tissues using NAS is comparable to the sequencing quality of reads generated from culture-derived DNA using both NAS and Illumina technologies. We present a new approach that provides complete and accurate genome sequence reconstruction, culture independently, and using an economically affordable technique.

## Introduction

Animal tuberculosis (TB) caused by *Mycobacterium bovis (M. bovis)* is a significant infectious disease affecting a wide range of domesticated and wild animals, representing a worldwide public health concern (Muller et al., 2013; Meiring et al., 2018). In South Africa, animal TB poses a serious threat to wildlife, including the iconic African buffalo (*Syncerus caffer*) (Bernitz et al., 2019). These animals serve as a reservoir for *M. bovis* and can transmit the pathogen to domestic cattle and other susceptible species (Sichewo et al., 2019). The implications of *M. bovis* infection in wildlife extend beyond the health of individual animals, as it can have far-reaching consequences for conservation efforts, public health, and the livestock industry (Muller et al., 2013).

Efficient and accurate surveillance and management strategies are crucial for mitigating the impact of animal TB in wildlife populations (Ortiz et al., 2021; Perea et al., 2021). Currently, detection and characterization of *M. bovis* has relied on culture-based methods, where bacterial isolates are obtained from infected animals and subjected to phenotypic and genotypic analyses (Goosen et al., 2022). However, this approach has inherent limitations, particularly when dealing with wildlife populations in general and foot-and-mouth virus endemic regions where movements of animals or samples are not permitted (NAHF, 2022). In addition, the isolation and culture of *M. bovis* from wildlife samples are challenging due to the complex microbiota and the presence of non-culturable or fastidious strains (Bull et al., 2016; Goosen et al., 2022). Moreover, the culture step is time-consuming and labor-intensive, impeding real-time surveillance and response (Perea et al., 2021).

In recent years, whole-genome sequencing (WGS) has revolutionized the field of infectious disease genomics, offering unprecedented resolution and insights into pathogen biology, transmission dynamics, and antimicrobial resistance (Meehan et al., 2019). WGS enables the comprehensive characterization of bacterial genomes, including the identification of strain types, the detection of genetic variations, and the prediction of drug resistance profiles (Rossi et al., 2023). In the context of *M. bovis*, WGS has proven invaluable in understanding the transmission patterns and evolution of the pathogen, guiding control measures, and investigating outbreaks (Rossi et al., 2023).

In South Africa, the implementation of *M. bovis* WGS in wildlife populations holds great promise for improved understanding of animal TB epidemiology and management. By obtaining high-quality genomic data directly from infected animals, it becomes possible to overcome the limitations of culture-based methods and obtain a more accurate representation of *M. bovis* diversity and population structure (Martin et al., 2022; Su et al., 2023). Furthermore, WGS allows for the identification of specific genetic markers associated with virulence, host adaptation, and antimicrobial resistance, providing valuable insights for targeted interventions (Pan et al., 2011).

This study explored a novel sequencing approach that has the potential to address and bridge the existing knowledge gap by comparing two next-generation sequencing (NGS) platforms for *M. bovis* detection and characterization in South African buffalo populations. The generation of whole-genome sequences for transmission and surveillance studies of animal TB based on culture extracts can be impeded by coinfection or contamination of samples with other microorganisms that outcompete the target agents causing animal TB (Maiga et al., 2012; Riello et al., 2016; Gopalaswamy et al., 2020). Co-infections can also modulate disease outcomes, particularly in infections with closely related microorganisms that share antigenic properties and mimic immunological escape mechanisms (Gorsich et al., 2018). Recent studies have demonstrated the advantages of using full-length 16S rRNA amplicon sequencing for taxonomic classification, as opposed to short-read amplicon sequencing (Earl et al., 2018; Callahan et al., 2019; Johnson et al., 2019). Nonetheless, previous methods for high-throughput full-length 16S rRNA gene amplicon sequencing using Oxford Nanopore Technologies (ONT) often relied solely on reference database alignment (Curry et al., 2022; Zorz et al., 2023) rather than employing *de novo* generation of sequence features, such as ASVs (amplicon sequence variants) or OTUs (operational taxonomic units). A major drawback for ONT has been its base-calling error rate. However, the recently released sequencing chemistry and base-calling software have been significantly improved, placing ONT as an attractive and reliable alternative to the Illumina platform (Oude Munnink et al., 2020; Smith et al., 2021). By using reference-free bioinformatic analyses of multiple housekeeping genes amplified using target specific primers on the ONT platform, we aimed to detect the presence of microbial contamination, including non-tuberculous mycobacteria (NTM), in each sample (Macovei et al., 2015; Vierstraete and Braeckman, 2022). We compared the performance of Illumina and Nanopore adaptive sampling (NAS) WGS on cultured extracts and NAS directly on DNA extracted from tissue homogenates. This study aimed to assess the accuracy and resolution of each method and explore the potential of NAS combined with an appropriate data analysis pipeline as a culture-independent alternative for comprehensive genomic surveillance studies. Based on these data, the ability of the NAS approach to distinguish target sequences from host genomic material was evaluated.

This research presents novel culture-independent, long-read based, and more accessible NGS approaches for a deeper understanding of the epidemiology and transmission dynamics of animal TB. The findings will inform evidence-based strategies for disease management, risk assessment, and control measures in wildlife populations, with implications for both animal health and human health. Furthermore, this study showcases the value of WGS as a powerful tool in wildlife disease surveillance, highlighting its potential for improving conservation efforts, preventing zoonotic transmission, and safeguarding the livestock industry.

## Methods

### Sample collection and culture

**Figure 1:**
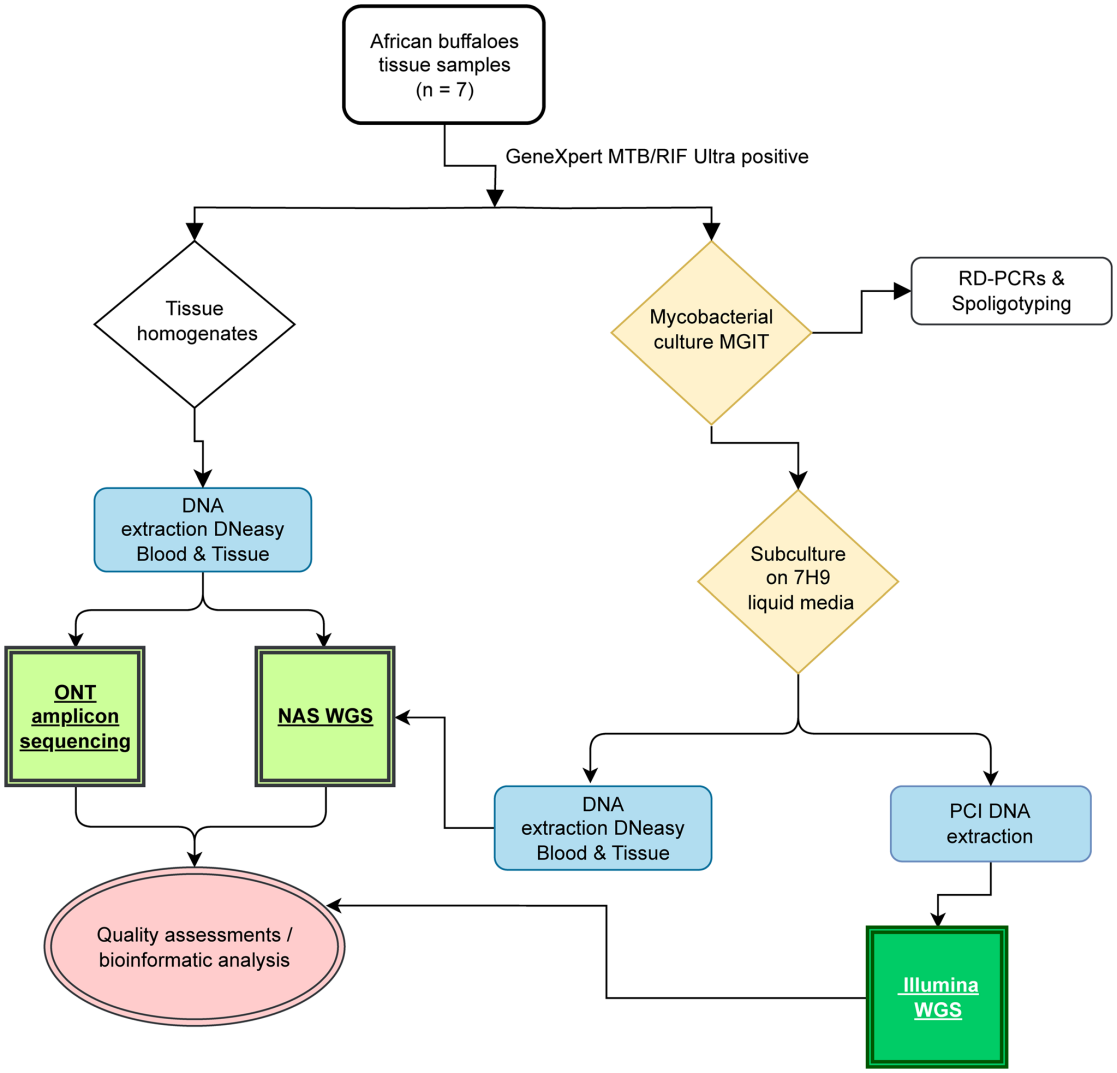
Study overview highlighting the key steps for long- and short-read based whole-genome sequencing and culture-independent deep sequencing of housekeeping genes. The workflow applied in the present study is reported, including tissue sample processing, mycobacterial culture (yellow), DNA extractions (blue), Illumina-based short read sequencing (dark green), Oxford Nanopore adaptive sampling (NAS), and Oxford Nanopore targeted amplicon sequencing (light green).

In 2018, a *M. bovis* test-and-slaughter program was conducted in Hluhluwe-iMfolozi Park (HiP), South Africa (SA), on a herd of African buffaloes (Bernitz et al., 2020; Smith et al., 2021). All animals were captured, immobilized and whole blood collected as previously described (Parsons et al., 2011). All animals of the herd (*n* = 50) were euthanized due to their high cell-mediated immunological (CMI) responses towards *M. bovis* specific antigens (>50% of the animals). Various tissue samples (head, thorax, lung lymph nodes and lung tissue) were collected during in-depth necropsies and lesion scores captured as previously described (Table 1; Bernitz et al., 2019). Mycobacterial cultures were performed on all samples using the BACTEC™ MGIT™ 960 Mycobacterial Detection System (Becton Dickinson). For each sample, parallel uncultured tissue homogenate aliquots were obtained. In brief, approximatively 10 g of tissue were homogenized in 50-mL skirted tubes (Becton Dickinson) containing eight 4.8-mm metal beads and 4 mL of sterile PBS using a blender (Bullet Blender 50; Next Advance) for 15 min at maximum speed as previously described (Goosen et al., 2022). The homogenates were subsequently preserved (− 80°C) for subsequent DNA extractions, repeat culturing and/or future sequencing. All culture-positive culture crude extracts were subjected to speciation using a PCR targeting genetic regions of difference to confirm *M. bovis* infections, as previously described (Warren et al., 2006). Further genetic speciation using spoligotyping was performed (Belakehal et al., 2022). Thereafter, 1 mL of each *M. bovis*-positive MGIT was further inoculated into 5 mL Middlebrook 7H9 media containing sodium pyruvate and incubated for an additional 21 days at 37°C for DNA extraction (Figure 1).

**Table 1:** Metadata of the seven African buffaloes included in the present study.

					Mycobacterial culture		Culture-independent PCR	
**Culture ID**	**Tissue type**	**Lesions score**	**Age**	**Gender**	**Final culture RD-PCR result**	**Spoligotyping**	**Tissue - GeneXpert MTB/RIF ultra**	***IS*6110/*IS*1081 Ct-values**
NB18193	Lung	2	Adult	Female	*M. bovis*	SB1474	MTB DETECTED HIGH	15.8
NB18254	Retropharyngeal	2	Juvenile	Female	*M. bovis*	SB1474	MTB DETECTED HIGH	15.1
NB18212	Lung	2	Juvenile	Female	*M. bovis*	SB1474	MTB DETECTED LOW	22.5
NB18191	Parotid	1	Adult	Female	*M. bovis*	SB1474	MTB DETECTED MEDIUM	16
NB18211	Tonsil	1	Adult	Female	*M. bovis*	SB1474	MTB DETECTED MEDIUM	16.8
NB18246	Pooled head	0	Juvenile	Female	*M. bovis*	SB1474	MTB DETECTED HIGH	15.6
NB18249	Lung	3	Adult	Female	*M. bovis*	SB1474	MTB DETECTED MEDIUM	16.3

Sample source, speciation using conventional molecular methods, and results of GeneXpert MTB/RIF Ultra applied on tissue homogenates is reported.

### Sample selection

For this pilot study, frozen native tissue homogenates from seven African buffaloes were retrospectively selected for downstream analysis. Selection criteria were based on the (1) quality of whole-genome sequences generated using the Illumina MiSeq instrument from culture, (2) tissue types, (3) lesion scores, and (4) varying amounts target genomic DNA. In detail, seven culture extracts obtained from tissue homogenates and sequenced with Illumina were selected based on the sequencing quality (coverage >99%, mean depth > 60X, and > 99% of mapped reads to the reference genome). In total, 3 lung samples, 1 retropharyngeal, 1 tonsil, 1 parotid, and 1 pool of the head lymph nodes presenting different lesion scores ranging from 0–3 were included (Bernitz et al., 2019; Table 1). The amount of *M. bovis* genomic DNA present in each native tissue homogenate was assed based on insertion elements *IS6110* and *IS1081* (combined detection) detected by Cepheid’s GeneXpert MTB/RIF Ultra (Ct values: 15.1–22.5), as previously described (Hlokwe and Mogano, 2020). Additionally, for each sample selected, a corresponding aliquot of the crude 7H9 mycobacterial culture was included for DNA extraction and NAS whole-genome sequencing alongside the corresponding native tissue homogenate.

### Whole-genome sequencing processing

#### Illumina platform

Genomic DNA was extracted from heat-inactivated (98°C for 45 min) culture pellets and WGS was performed as previously described (van Soolingen et al., 1991). Briefly, libraries were prepared with the Nextera DNA Flex Library Prep Kit (Illumina). The quality of each library was assessed with a FragmentAnalyzer Automated CE System (Agilent) using a Next Generation Sequencing Fragment Kit (1–6,000 bp; Agilent). Libraries were paired-end sequenced in 250 bp reads on an Illumina MiSeq System using the MiSeq Reagent Kit (version 3, 600-cycle)(Ortiz et al., 2021).

#### Oxford Nanopore

Total DNA was extracted using a modification of the DNeasy Blood and Tissue kit (Qiagen) directly from (a) 1 mL uncultured tissue homogenate aliquots (*n* = 7) and (b) paired 1 mL 7H9 crude mycobacterial culture pellets (*n* = 7) as previously described (Stanton et al., 2010). In brief, subsequently to heat-inactivation (98°C for 45 min) and centrifugation (1,500 x *g* for 10 min), 300 μL of Buffer ATL was added to the cell pellet. A total of 25 μL Proteinase K was added and left for digestion at 56°C at 600 rpm in a thermo mixer overnight. The solution was centrifuged at 5,500 × g for 5 min, 500 μL of the supernatant recovered, and transferred to a 1.5 mL tube. Thereafter, 400 μL of buffer AL and 400 μL of ethanol were added to the solution and transferred to a Mini Spin Column. Finally, the exposed DNA was purified using washbuffers AW1 and AW2 and eluted in 60 μL of buffer AE prewarmed at 54°C. DNA concentrations were determined using the Qubit 1x dsDNA High Sensitivity Assay kit (Thermo Fisher Scientific), according to the manufacturer’s instructions (Supplementary Table S1).

#### Nanopore adaptive sampling

**Figure 2:**
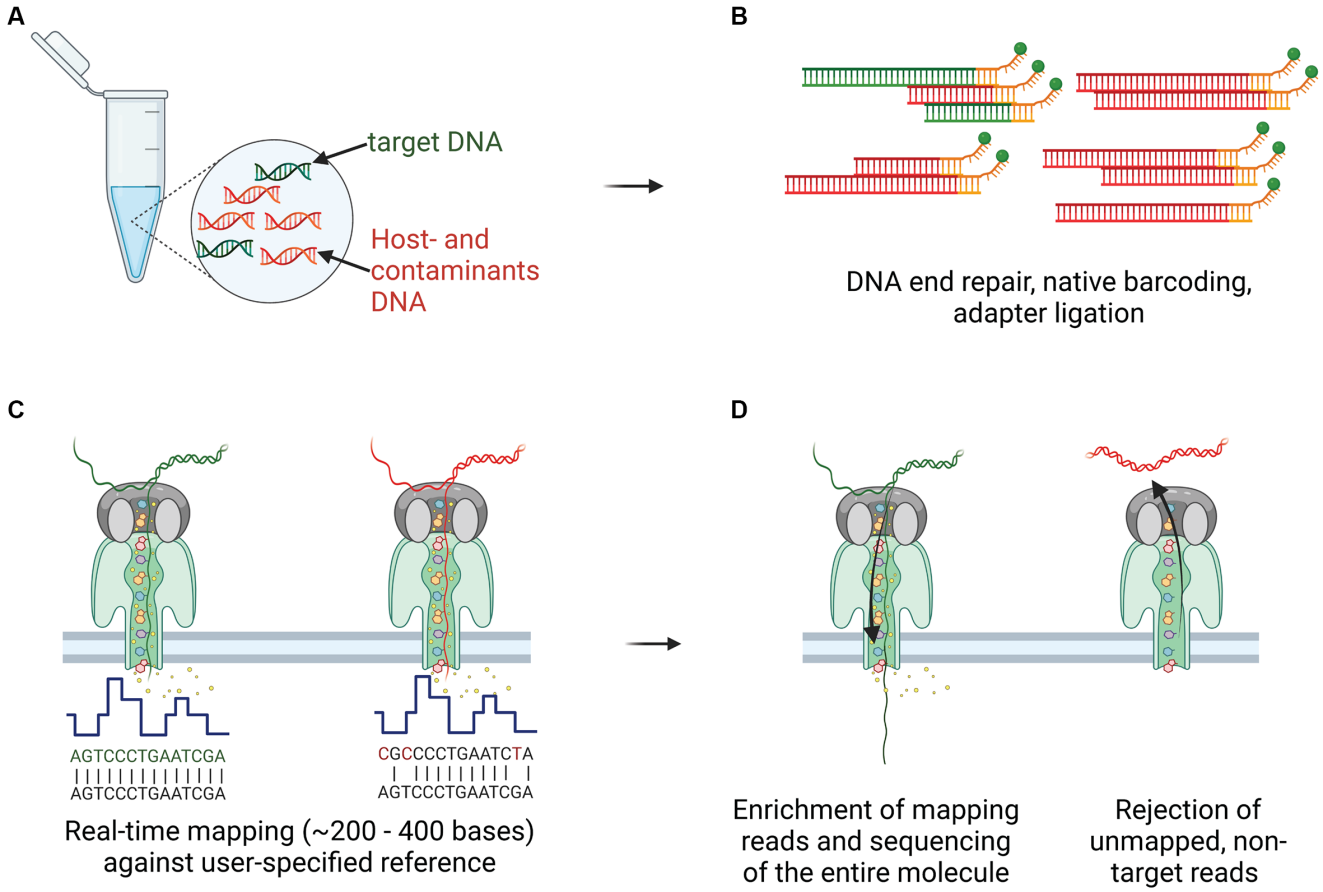
Culture-independent, long-read sequencing of *M. bovis* genome in a clinical sample using Nanopore adaptive sampling (NAS). **(A)** Target gDNA (green) mixed with various amounts of host- and contaminant genomic material (red), was extracted from homogenized tissue samples, containing macroscopic TB lesions. **(B)** Library preparation, including DNA ends repair and dA-tailed, native barcoding and ligation of the adapters to the DNA fragments, was performed before transferring to the flow cell for sequencing. **(C)** Real-time mapping of nucleotide sequences (between 200 and 400 bases) against a user-specified reference (*M. bovis* AF2122/97). **(D)** Target gDNA molecules were enriched and sequenced throughout their entire length, whereas non-target reads not mapping to the reference were rejected after pore voltage inversion.

The DNA from culture-independent tissue homogenates and crude 7H9 *M. bovis* positive culture extracts from the same animals were all first diluted with EDTA (pH = 8) to a final concentration of 34 ng/μl. Thereafter, 12 μl (~400 ng) were FFPE repaired and end-prepped using NEBNext FFPE DNA Repair Mix and NEBNext Ultra II End Repair/dA-tailing module (New England Biolabs, Inc.). Each sample was individually barcoded using NEB Blunt/TA ligation (New England Biolabs, Inc.) and the Native Barcoding Kit 96 V14 (SQK-NBD114.96), as described by the manufacturer (ONT). Adapter ligations were performed on each barcoded DNA library using Quick T4 DNA Ligase in the NEBNext Quick Ligation Module and NEBNext Quick Ligation Reaction Buffer (New England Biolabs, Inc.). Thereafter, each DNA library was loaded onto separate R10.4.1 flow cells (>1,250 pores available, FLO-MIN114, LOT 11002667, SN B024009663), adaptive whole genome sequenced using reference genome *M. bovis* AF2122/97 (Accession NC_002945.4) for enrichment on the MinION Mk1C device for 22–27 h (Figure 2).

#### Detection of contaminants using ONT targeted amplicon sequencing

**Figure 3:**
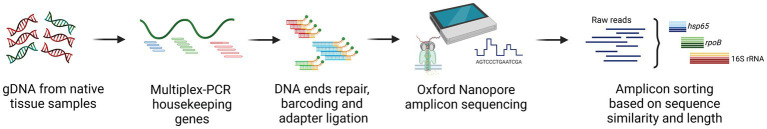
Overview of the culture-independent targeted amplicon sequencing approach using Oxford Nanopore Technologies (ONT) sequencing platform. After DNA extraction directly from tissue samples, ONT library preparation, including DNA ends repair, native barcoding and adapter ligation, was performed. The prepared library was loaded on the flow cell (R10.4.1) and inserted in the sequencer (MinION). Basecalling was carried out real-time, storing the data in FASTQ format. Raw reads were subsequently used for downstream bioinformatic analysis and enabled characterization and quantification of the bacterial community present in the samples.

The amplification and deep-sequencing of three housekeeping genes, namely *hsp65* (441 bp) (Telenti et al., 1993), *rpoB* (680 bp) and the full-length 16S rRNA gene (~1,500 bp) (Johnson et al., 2019), was performed culture-independently directly on DNA extracted from buffalo tissue homogenates as previously described (Clarke et al., 2022). For the *rpoB* gene, newly designed primers were applied (F1-CGTGTGTATGTGGCTCAGAA and R1-GTGTCATCGGACTTGATGGT). A 25 μL reaction contained 14 μL Q5 High-Fidelity 2X Master Mix (New England Biolabs, Inc.), 0.5 μL of each 50 μM primer stock solution, 6 μL sterile, nuclease free water and 2 μL undiluted extracted DNA. PCR cycling conditions were as follows: 1 cycle initial denaturation at 98°C for 15 min, followed by 40 cycles of denaturation (98°C for 30 s), annealing (62.5°C for 30 s) and elongation (72°C for 2 min). Final elongation took place at 72°C for 5 min. Presence of the amplified products was confirmed by 1% agarose gel electrophoresis. Thereafter, amplicons were end-repaired, individually barcoded, pooled into a single library, native adapter ligated, loaded onto a single R10.4.1 flow cell (>1,250 pores), and sequenced using the MinION mk1C device (Figure 3).

### Bioinformatics

#### Illumina and ONT whole-genome sequencing

Data analysis was applied to 7 datasets generated with the Illumina MiSeq and 14 datasets generated with MinION mk1C. For the NAS sequencing datasets, once the data acquisition was stopped after 22–27 h (Table 2), Guppy [v6.4.6] was used for basecalling (260 bps – High-Accuracy), demultiplexing and trimming of the barcodes (Wick et al., 2019; Steinig and Coin, 2022). Reads with a Q score of <8 were discarded. Quality control, filtering, and summary reports for nanopore reads were generated using nanoq v 0.10.0 (Wick et al., 2019; Steinig and Coin, 2022). The quality of sequencing reads was evaluated using FastQC (Version 0.11.9)^1^ and pycoQC (v2.5.0.23)^2^ (Leger and Leonardi, 2019). Thereafter, sample contamination was evaluated via taxonomic classification using Kraken2 (v2.1.1)^3^ (Wood et al., 2019).

**Table 2:** Overwiev of Nanopore adaptive sampling (NAS) sequencing runs and basecall summary.

	**NAS direct**	**NAS culture**
Estimated bases	2.75 Gb	2.42 Gb
Data produced	42.41 GB	44.77 GB
Reads generated	579.85 k	1.64 M
Estimated N50	6.07 kb	4.97 kb
Elapsed time	22 h 20 min	27 h 18 min

Genome sequences generated with both platforms were assessed with the validated vSNP3 pipeline^4^ tool of the US Department of Agriculture-Veterinary Services (Supplementary material S1). The evaluation of each isolate’s sequencing run performance was carried out using a synopsis of quality metrics generated with default settings. Finally, sequencing coverage and mapping quality across the reference genome was performed in QualiMap [v.2.2.2-dev] and visualized in GraphPad Prism (v10.0.0) (Parsons et al., 2011; Smith et al., 2021). After performing various filtering steps to remove error prone region variant positions (Supplementary material S1), informative SNPs were used to create SNPs tables and phylogenetic trees. Single indels’ accuracy was manually confirmed on an isolate-by-isolate basis using bam files generated in Step 1 and visualized in Artemis [v18.1.0]. Only validated SNPs were retained for downstream analysis. The validated SNPs were used to generate an SNP alignment file, which was utilized to build a maximum likelihood phylogenetic tree using RAxML software [v8.2; GTR CAT model] (Stamatakis et al., 2005). To confirm the assigned *in silico* spoligotypes and determine the animal lineage of the sequences, the TBprofiler pipeline using H37Rv as a reference was used to analyze short- and long-reads using the flag –platform Illumina and ONT, respectively (Phelan et al., 2019).

To evaluate the quality of the genome mapping, two parameters were monitored: (i) genome completeness, and (ii) sufficient sequencing depth. To assess the first parameter, the percentage of the total number of covered positions of the reference sequence (*M. bovis* AF2122/97) was determined after mapping with BWA and Minimap2. For the second parameter, the percentage of genome positions with a sequencing depth below 20X, defined as N%, was retrieved from the bam files using BEDTools (Quinlan and Hall, 2010) in accordance with previous minimal sequencing depth observations. Comparison of mean numbers of no coverage bases between Illumina, ONT direct and ONT from culture was assessed using multiple unpaired t-test (GraphPad Prism 10.0.0).

Ultimately, to conduct a comparison between the acquired whole genome sequences produced using the ONT platform and sequences from various *M. bovis* strains generated through the same method, the US National Center for Biotechnology Information (NCBI) was searched for publicly available genomes. The following search terms were applied: “*Mycobacterium tuberculosis* variant *bovis* [organism]” AND “Nanopore.” A total of 98 genome sequences concordant with our search terms were available (1st of August 2023). From these genomes, we excluded those that met the following criteria prior to downstream analysis: genomes registered as bacillus Calmette-Guérin (BCG) and average whole-genome coverage <15X. One genome sequence from *M. bovis* BCG and *M. tuberculosis* H37Rv each were included as outliers. The retrieved sequences were subsequently analyzed using the vSNP3 pipeline as described above and the acquired phylogenetic tree was visualized using iTOL (v5) (Letunic and Bork, 2021). Pairwise SNP distance matrixes from SNP alignments were computed using snp-dists (version 0.8.2)^5^ and used to draw heatmaps combined with the corresponding dendrograms using the pheatmap package.^6^

In conjunction with the vSNP3 pipeline, MINTyper (Hallgren et al., 2021), designed to compare sequences from both the Illumina and ONT platform, were implemented to determine genetic distance between the sequences and estimate a distance matrix.

#### Distribution analysis of insertion sequences IS*6110* and IS*1081*

The ISMapper (v2.0) pipeline (Hawkey et al., 2015) was applied on genome short-read sequences for identification of IS*6110* and IS*1081* using the AF2122/97 reference genome. The identified genomic positions were subsequently manually checked for their completeness using the bam files and Artemis (v18.1.0). Once the presence and location of the ISs were confirmed, an *in silico* PCR with previously published primers was performed (CLC Genomics Workbench v22; Qiagen) to estimate the quantity of genomic DNA load present in the native material used for adaptive sampling and as culture inoculum.

#### Detection of contaminants using ONT targeted amplicon sequencing

For targeted amplicon sequencing, base-calling was performed in real-time using Guppy [v6.4.6] (Wick et al., 2019) and the 400 bps – Low-Accuracy option. Data acquisition and base-calling were stopped after 23 h. Quality control, filtering, and summary report were performed as above. Reads with a Q score of <12 were discarded. Thereafter, reference-free reads sorting, based on similarity and length, was performed using the amplicon sorter tool [v2023-06-19] (Vierstraete and Braeckman, 2022). A total of 100,000 randomly chosen reads with minimum and maximum lengths of 300 bp and 2000 bp, respectively, were selected for each barcode. Consensus sequences were grouped for each species and amplified target gene, and relative abundances were retrieved based on the representative pool of reads analyzed (Figure 3). Finally, a reference database for Mycobacteria was built using the nsdpy python script.^7^ Briefly, for each target gene, the nsdpy (NCBI sequence Downloader) was used to retrieve publicly available sequences using the following commands nsdpy -r “Mycobacteriaceae[Organism] AND gene_of_interest[Title]”; with gene_of_interest defined as *hsp65*, *rpoB*, and 16S rRNA, respectively. The downloaded sequences were used to build a database for each gene using ABRicate^8^ and the same tool was applied for the screening of consensus sequences and summarizing the report files. The distribution of consensus sequences generated per target gene was visualized using the R package ggplot2.

For all bioinformatics tools, we used the default settings unless stated otherwise.

## Results

All seven buffaloes sampled in the present study were confirmed to be *M. bovis* infected and the presence of the *M. bovis* region of difference 4 (RD4) signature was confirmed from culture positive crude extracts (Warren et al., 2006). Spacer oligonucleotide typing hybridization assay (spoligotyping) revealed profile SB1474 for all the tested samples, based on mycobacterial culture extracts. Results from the GeneXpert MTB/RIF Ultra, using tissue homogenates, revealed the presence of MTBC genomic DNA in every sample tested, at low (n = 1), medium (n = 3) and high (n = 3) relative concentrations (Table 1).

### Comparison of Illumina, direct and culture derived NAS sequences using vSNP3 and MINTyper pipelines

**Figure 4:**
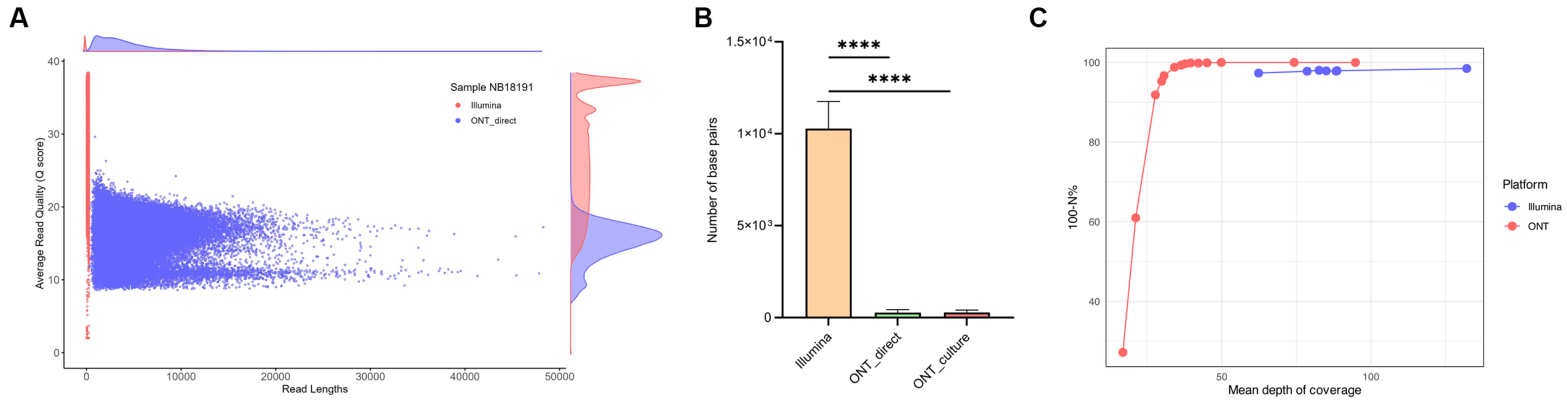
Coverage and genome quality across the reference, comparison of culture-derived Illumina reads with culture-derived and culture-independent reads generated with Nanopore adaptive sampling (NAS). **(A)** Distribution of read lengths and average quality (*Q* values) for Illumina and culture-independent data generated with NAS for sample NB18191. **(B)** The number of bases with no coverage (depth = 0) was compared between the three groups. The Illumina dataset showed the highest number of bases with no coverage with 0.24% (*M* = 10,279 positions, SD = 1,369.24), followed by the two ONT datasets with similar results and 0.006% of the reference genome not being covered (ONT culture *M* = 275 positions, SD = 126.2 and ONT direct *M* = 271 positions, SD = 148.73) (****p < 0.0001). **(C)** Comparison of mean depth of coverage for *M. bovis* genomes and their quality at single position level. The number of positions with a sequencing depth below 20X (N%) was compared with corresponding mean depth of coverage over the entire genome.

The results of the sequencing comparison among the three groups of DNA sequencing sources (ONT direct and ONT culture – sequences generated using NAS from DNA extracted from tissues and crude liquid culture, respectively, and Illumina – sequences generated from DNA extracted from the same liquid culture as ONT culture) are presented in Supplementary Table S1. The Illumina platform generated the highest mean read count with approximately 1,682,963 reads, followed by the ONT direct group with a mean read count of 47,105, and the ONT culture group with the lowest mean read count at 42,229 using comparable amounts of DNA and similar multiplexing. The average read length of the NAS generated reads was 3,470 bp and 4,648 bp for the ONT culture and ONT direct groups, respectively, whereas the mean size of the longest reads per each group was 58,177 bp for the ONT culture derived reads and 50,928 bp for the ONT direct group. Both the ONT direct and ONT culture groups showed similar mean values for passing Q20 (≥ 77%) and Q30 (≥ 51%). Sequences generated using the Illumina platform had higher mean values for passing Q20 (94.01%) and Q30 (90.27%) and demonstrated the highest mean read quality with a value of 31.4, while the ONT groups had a mean read quality of 16.9 from culture and 16.4 directly from tissue as shown in Figure 4A for sample NB18191 and in Supplementary Table S1.

The Illumina reads exhibited a significantly (*p* < 0.0001) lower mean reference genome coverage of 99.76%, compared to mean coverage of >99.99% for ONT culture and ONT direct groups (Figure 4B and Supplementary materials S2, S3). In other words, the Illumina group had the highest number of bases with no coverage (depth = 0) with 0.24% (*M* = 10,279 positions, SD = 1,369.24), followed by the two ONT datasets with similar results and only 0.006% of the reference genome not being covered (ONT culture *M* = 275 positions, SD = 126.2 and ONT direct *M* = 271 positions, SD = 148.73) (Figure 4B). Mean depths of coverage for Illumina were 62X - 132X, followed by the ONT direct group with means of 27X - 74X, and the ONT culture group with mean depths of coverage of 16X - 49X. For ONT generated sequences, high-quality genomes (defined as >99% of genomic positions with a sequencing depth above 20X) were retrieved from data showing at least 36X mean depth of coverage (Figure 4C). The number of positions showing the minimal sequencing depth (defined as 20X) dropped significantly with mean depth of coverage below 30X, showing only 91 and 60% of positions fulfilling the criteria in genomes with 27X and 21X mean depth of coverage, respectively. For the Illumina data, the number of genome positions with minimum sequencing depth was very stable, independently from the mean depth of coverage, and ranged between 97.3–98.4% (Figure 4C).

The obtained sequences were classified as spoligotype SB1474, belonging to the EU1 clonal complex, group Mbovis-09 (Supplementary Table S1). The *in silico* spoligotyping result was confirmed using the TBprofiler pipeline, which additionally defined all the sequences as animal lineage La1.8.1 (Zwyer et al., 2021).

A total of 11 variants discriminating between the 21 analyzed genomes were identified using vSNP3 pipeline (Supplementary Table S2). These were manually inspected and excluded from the comparison due to the following reasons. The first SNP reported by vSNP3 was located within a 30-base deletion between positions 1,122,168 and 1,122,198 of the reference strain (Supplementary material S4). All reads from both platforms were inaccurately mapped in this region. Another large deletion of 147 bases, located between positions 3,082,830 and 3,082,977, led to two incorrect variant calls, defined as “A,” “T,” “C,” “G,” and “N.” The pipeline incorrectly assigned variant nucleotides in 3 out of 7 genomes for Illumina mapped reads. For long reads mapped to the reference genome, only 9 out of 28 calls were correctly assigned as deletions. At position 4,194,117, listed as a putative transposase gene, 6 erroneously assigned variants were observed for the mapped short reads, while 14 correct nucleotide assignments were made for the ONT generated reads. Additionally, the remaining 7 variants were in PPE and PKS encoding genes and were excluded from the comparison due to known difficulties with short reads in resolving these genomic regions. Upon manual investigation of the reported variants compared to the reference genome, all 98 reported SNPs from the 14 ONT sequences were found to be correct. Overall, the 7 genomes were defined to be clonal and by including comparable positions using short- and long-reads data mapped over the entire genome, no SNPs differences were retrieved. Outputs from the MINTyper analysis were in concordance with the vSNP3 analysis and a SNP difference of 1 was detected between the Illumina and ONT sequences.

**Figure 5:**
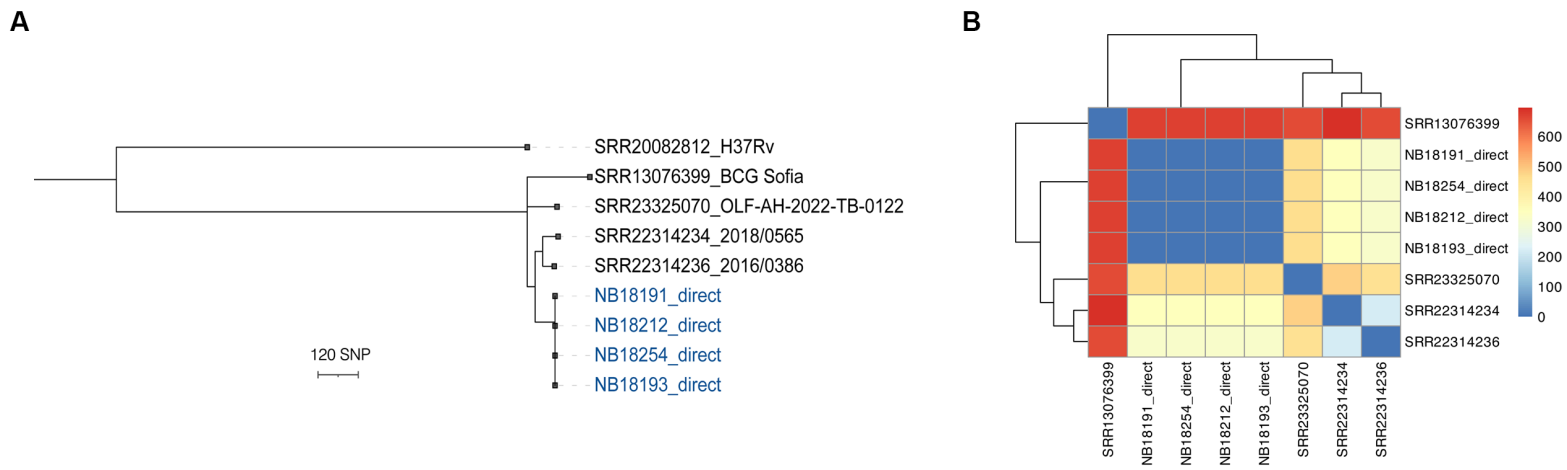
Phylogenetic analysis with publicly available *M. bovis* strains, one *M. bovis* BCG strain and four representative genomes from the study generated culture-independently using NAS. **(A)** Maximum likelihood tree was constructed with RaxML and rooted with an H37Rv *M. tuberculosis* genome generated with ONT. The scale bar represents a branch length of 120 SNPs. **(B)** Heatmaps of pairwise comparison matrix were derived from SNP distances combined with the corresponding dendrograms for *M. bovis* and *M. bovis* BCG sequences.

Finally, using publicly available sequences generated with the ONT platform, the ability of the vSNP3 pipeline to compare long-read data for transmission investigation purposes was evaluated. A summary of the quality metrics for the retrieved genomes is provided in Supplementary Table S3. Mean depth of coverage ranged between 23X and 1,949X. The three *M. bovis* genomes obtained from clinical isolates showed distinct profiles when subjected to *in silico* spoligotyping (SB0971, SB0673, and SB0130). The pipeline was able to accurately retrieve a total of 3,508 discriminatory SNPs compared to the reference sequence (Supplementary Table S4). In the phylogenetic tree, the clonal position of four selected *M. bovis* genomes from African buffaloes was confirmed, and a clear distinction between these sequences and the publicly available sequences was evident (Figures 5A,B).

### Distribution analysis of insertion sequences IS*6110* and IS*1081*

By analyzing the occurrence of insertion sequences (IS) IS*6110* and IS*1081*, we were able to determine that all genomes contained a single copy of IS*6110* and six copies of IS*1081,* respectively. Of the latter six copies, one copy was truncated (Charles et al., 2023) and not amplifiable using the GeneXpert MTB/RIF Ultra primers. The genomic positions of the IS were conserved across the sequences analyzed. Similarly to *M. bovis* BCG for which an equal number of IS*6110* and IS*1081* copies was described (Chakravorty et al., 2017), we estimated a detection limit for GeneXpert MTB/RIF Ultra of 143.4 CFU/mL (95% CI, 106.2 to 243.7 CFU/mL).

### Detection of contaminants using ONT targeted amplicon sequencing

**Figure 6:**
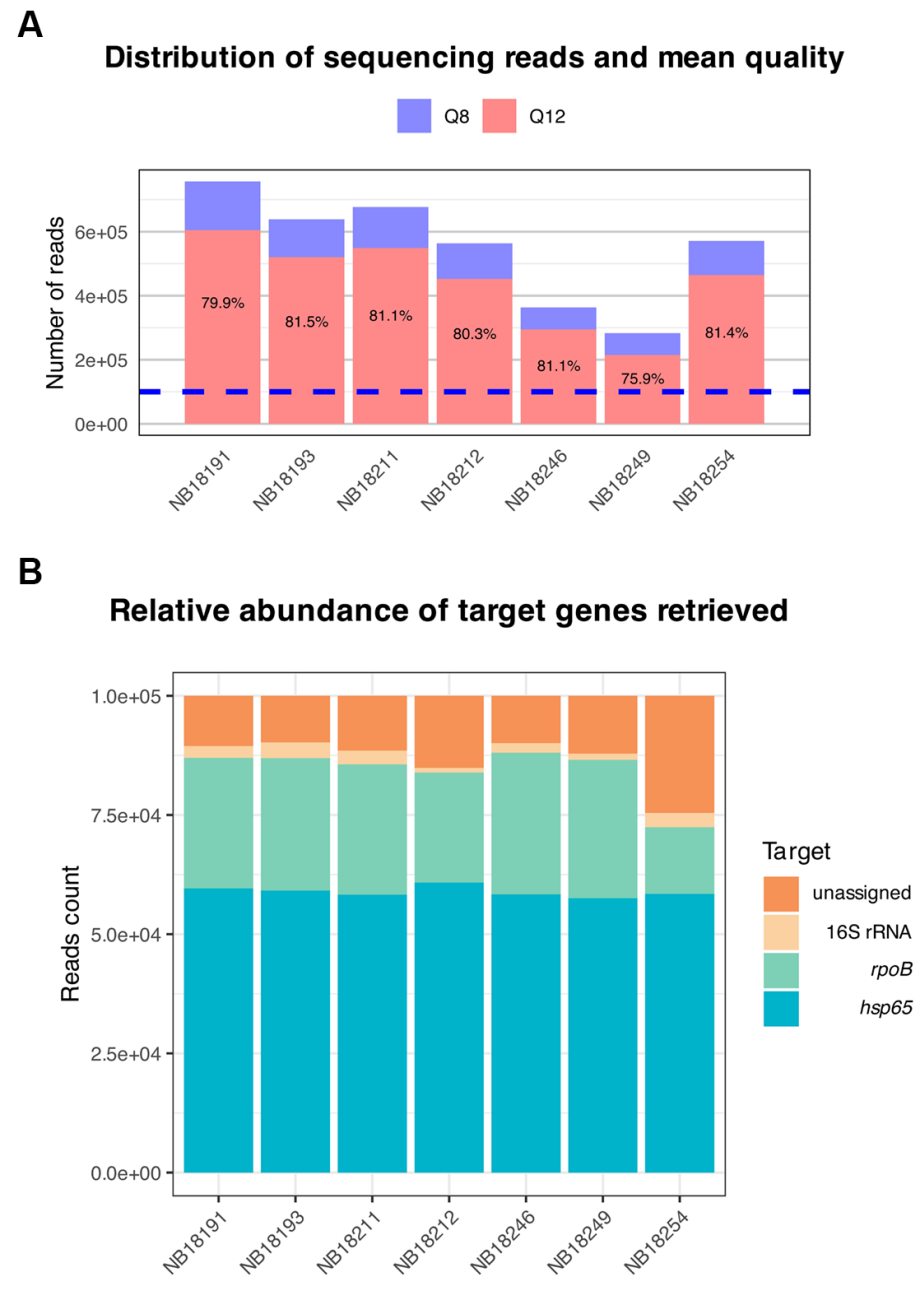
Overview of the targeted amplicon sequencing output. **(A)** Distribution of sequencing reads and mean quality. Raw reads basecalled with Guppy were automatically filtered at Q8 (blue). Subsequently, approximately 80% of the reads were retained after filtering with nanoq at Q12 (red). Of these, a total of 100,000 reads (dotted blue line) were randomly selected for downstream analysis with the amplicon sorter pipeline. **(B)** Reference-free sorting and assembly of consensus sequences were performed on randomly selected reads. The relative abundance of the target genes amplified using a multiplex PCR and sequenced using Oxford Nanopore Technologies is shown.

The *hsp65, rpoB*, and 16S rRNA gene regions were successfully amplified for each sample (Table 3). The number of reads with Q8 ranged from 283,064 to 756,804 (*M* = 550,441 reads, SD = 157,423), and the total number of bases sequenced ranged from 172,922,714 to 464,220,866 (*M* = 339,459,209 bases, SD = 97,863,859). The N50 read length, which represents the median read length of the longest contigs, varied from 563 to 568 bases. The mean read length for the samples ranged from 572 to 639 bases. The mean read quality, as indicated by the Phred score, was consistently above 13.0 for all samples. After quality-filtering the raw reads at Q12, the number of reads was reduced by 80.5%. Of these, 100,000 reads showing a mean quality of 14.3 (SD = 0.07) were randomly selected for downstream analysis (Figure 6A). This mean quality threshold was chosen due to the usually sterile nature of these body sites and the significant computational resources required to analyze a larger number of reads without additional informative value.

**Table 3:** Summary of data output for targeted sequencing from PCR-derived amplicons.

Culture ID	*hsp65*	*rpoB*	16S rRNA	Number of reads	Number of bases	N50 read length	Mean read length	Mean read quality
NB18193	+	+	+	638,707	408,202,430	568	639	13.7
NB18254	+	+	+	570,988	359,167,245	568	629	13.73
NB18212	+	+	+	563,289	322,558,743	563	572	13.65
NB18191	+	+	+	756,804	464,220,866	565	613	13.6
NB18211	+	+	+	676,936	419,171,718	566	619	13.68
NB18246	+	+	+	363,303	229,970,748	567	632	13.64
NB18249	+	+	+	283,064	172,922,714	567	610	13.32

“+” successful PCR amplification.

One consensus sequence per target in each sample was generated. Consensus sequences for *hsp65* were obtained after combining >31,000 reads per sample (*M* = 55,024 reads, SD = 9,706), >23,000 (*M* = 27,337 reads, SD = 1963) for *rpoB*, and between 980 and 3,309 reads (*M* = 2,279 reads, SD = 815) for the 16S rRNA gene (Figure 6B). Finally, consensus sequences were queried against curated databases generated for each gene and the output was evaluated for sequence coverage and percentage identities. All consensus sequences matched to reference sequences from the MTBC for the respective genes with coverage and identity of above 99.9 and 99.7%, respectively (Supplementary Table S5), excluding the presence of additional microorganisms, including other *Mycobacterium* spp.

## Discussion

Whole-genome sequencing (WGS) has emerged as the gold standard for bacterial outbreak investigation and pathogen typing, offering a level of resolution that conventional molecular methods cannot achieve (Bogaerts et al., 2021). The continuous evolution of sequencing methods provided by platforms based on different techniques brings forth new possibilities and increased accessibility to data, particularly in under-resourced areas or limited research settings. By applying a culture-independent approach and comparing the results with conventional culture-based Illumina WGS, we were able to retrieve crucial data for epidemiological purposes. In South Africa, there is a lack of information regarding the transmission and spread of animal TB, especially in complex ecosystems such as Natural Parks where multiple susceptible animal species share the same habitat. By providing evidence-based data regarding the burden of animal TB to the competent authorities, the use of *in vivo* tests with increased sensitivity or vaccination of susceptible animal populations for TB control will be facilitated. Therefore, there is a need for tools that enable unequivocal characterization of *M. bovis* in food-and-mouth disease (FMD) affected areas where culture of suspicious tissues samples is not possible (Arnot and Michel, 2020; Davey, 2023). Not only conventional markers, including spoligotyping and MIRU-VNTR markers, were retrieved from long reads generated by the ONT MinION Mk1C device, but high-quality SNPs used for transmission investigation at the deepest level were obtained and compared with short-read data derived from the Illumina MiSeq instrument. We report the presence of a clonal strain of *M. bovis* appertaining to the Eu1 clonal complex and showing spoligotype SB1474. The latter is a commonly reported spoligotype in South African buffaloes and has been circulating among this wildlife species for at least 15 years, being reported for the first time in 2007, and added to the Mbovis.org spoligotype database in 2008 (Hlokwe et al., 2011). According to the new nomenclature for livestock associated MTBC ecotypes, the sequences were classified as La1.8.1, which is one of the eight described for *M. bovis* (Zwyer et al., 2021). These sublineages exhibit significant differences in their geographic occurrence, with some being confined to specific regions while others being prevalent worldwide, suggesting different adaptation abilities and possibly host predilections. La1.8.1 is one of the most well-studied clusters of *M. bovis* strains, which is known to be widespread in the UK and regions that have historical trading ties with the UK (Zwyer et al., 2021). Hence, it is likely that the strain was introduced to South Africa through infected livestock originating from the UK and subsequently spread to wildlife, particularly African buffaloes in this specific case. More data, however, are necessary to support this hypothesis.

The NAS, also known as selective sequencing, is a newly developed method that relies on software-controlled enrichment, providing a potential solution without physical manipulation. A programming interface called ReadUntil allows for precise control over individual nanopores, enabling the software to request the removal of the currently sequenced molecule from a specific pore (Martin et al., 2022; NASCarD, 2023). By examining the initial portion of a molecule, typically a few hundred bases, a determination can be made if the molecule is the desired “target” or not. If a molecule is deemed “off target,” the current across the pore is reversed, rejecting the molecule, and allowing the pore to capture a new one. It is crucial for this rejection process to occur quickly to ensure the molecule is ejected before a significant portion of it is sequenced. The speed at which decisions are made and rejections occur directly impacts the potential level of enrichment achievable (Martin et al., 2022).

Short-read sequencing exhibits a pronounced bias towards GC content, but it excels in accurately detecting single nucleotide variants and small indels (Chen et al., 2013). In contrast, long-read sequencing offers the advantage of resolving structural variations and variants found within repetitive regions (Chen et al., 2023). For instance, the PE/PPE gene families in *M. tuberculosis*, which constitute around 10% of its coding regions, have been implicated in virulence and their association with drug resistance warrants further exploration (McEvoy et al., 2012; Dippenaar et al., 2022). Unfortunately, short-read sequencing struggles to accurately resolve these regions, resulting in their exclusion from bioinformatics studies on *M. tuberculosis*. However, long-read sequencing holds the potential to provide a more comprehensive understanding of these regions, including their impact on resistance phenotypes and strain pathogenesis. It is important to note that until recently, long-read nanopore sequencing faced accuracy limitations due to error-prone homopolymer regions (Dippenaar et al., 2022). The introduction of R.10 flow cells, specifically designed to optimize translocation speed for homopolymer sequences within pores, along with improvements in base-callers such as Guppy, has significantly enhanced sequence accuracy (Dippenaar et al., 2022). Consequently, variant calling and subsequent drug-resistance prediction have also improved.

Precise characterization of bacterial communities present in infected patients is crucial to understanding their diversity and potential role in the disease and can help identify microbial biomarkers, expected to be consistently enriched or depleted in patient cohorts (Duvallet et al., 2017). In this study, we investigated the capabilities of Nanopore sequencing to deliver high-accuracy housekeeping gene profiles for the genus *Mycobacterium*, along with *de novo* generated consensus sequences using the full-length 16S rRNA gene amplicon sequencing. Upon applying a deep-sequencing culture-independent approach on native DNA extracted directly from TB confirmed tissues, we excluded the presence of other microorganisms potentially interfering with NAS. The abundance distributions of amplicons obtained for the three target genes should be regarded with caution since the affinity of the primer pairs used may differ significantly. It is the authors’ opinion that, by implementing the outlined method, future studies on samples presenting multiple mycobacterial species and possibly from body locations presenting microbial colonization, such as the upper respiratory tract, will demonstrate the specificity of the NAS and facilitate a deeper understanding of the interactions and within-host coexistence of microbiome and TB causing agents.

Animal TB is difficult to control and eradicate in part due to the costly nature of surveillance and poor sensitivity of *ante*- and *post-mortem* diagnostic tests (Meiring et al., 2018). The disease has a significant impact on agriculture, biodiversity, and the financial situation of farmers, particularly in low-income countries. Detection of TB in livestock results in a loss of profit for the farm, mainly due to the animal’s slaughter and replacement (Perez-Morote et al., 2020). Recent studies have shown that animal TB causes increased mortality, reduced milk and meat productivity, reduced fertility, and organ or carcass condemnation (Tschopp et al., 2021). National and supranational surveillance and monitoring programs have reduced TB prevalence in cattle, but spillover at the livestock–wildlife–human interface represents a challenge for eradication and leads to pathogen maintenance, both in animals and in the environment (Fitzgerald and Kaneene, 2013).

According to the WHO tuberculosis laboratory biosafety manual, any manipulation of clinical specimens suspected of containing *M. tuberculosis* should be conducted in high-risk or TB-containment laboratories (WHO, 2012; Ssengooba et al., 2015). Depending on local and national regulations, similar biosafety restrictions are applied to mammal samples suspected to be infected with animal tuberculosis. Although the establishment and maintenance of TB-containment laboratories are financially challenging in low-income countries, the escalating prevalence of disease is expected to drive a higher need for these facilities (Ssengooba et al., 2015). Retrieving high-quality whole-genome sequences from native material (independent of culture), will not only shorten the necessary time for data generation and subsequent implementation of restriction measurements but will also provide accessibility to crucial data in resource-limited settings, without the need for establishing the costly biosafety laboratory settings necessary for culture and manipulation of MTBC members. It is important to note, however, that manipulation of samples suspected to be infected with *M. bovis*, and especially procedures that lead to aerosol formation should be undertaken using the necessary biosafety precautions, including inactivation steps at the initial stage of DNA extraction. Finally, the risk of infection for laboratory personnel associated with MTBC culturing will be significantly lower.

Currently, there are no established recommendations regarding the minimum coverage threshold for whole-genome sequences obtained from long-reads for investigating transmission and outbreaks. According to the literature, it is suggested that a minimum threshold of 10X-30X coverage for short-read generated sequences is required to identify SNPs associated with antimicrobial resistance (AMR) in *M. tuberculosis* (Ellington et al., 2017; Bogaerts et al., 2021). We investigated the effect of a 20X threshold on short- and long-read generated sequences at a single position level, in order to determine the number of “high-quality” positions over the entire genome. For this study, and because of the small number of available genomes, all obtained sequences were analyzed using the vSNP3 pipeline and included in downstream analysis. In the future, to more accurately decide whether to exclude or include a genome for downstream analysis and transmission investigation, additional data are required to further evaluate the accuracy of the ONT platform. When comparing the number of genomic positions covered with a minimum sequencing depth of 20X between genomes generated using the Illumina and ONT platforms, it became evident that, regardless of the mean coverage depth achieved, short-read data from the Illumina platform cannot match the reference genome coverage achieved by long-read data, likely due to the inability of short reads to resolve repetitive regions of the genome.

Finally, for the purpose of this pilot study, only samples originating from presumably sterile tissue locations were included. Based on previous semiquantitative measurements using GeneXpert MTB/RIF Ultra (Chakravorty et al., 2017), different amounts of genomic DNA were detected in the tested samples, varying from low to high bacterial loads and corresponding to an estimated 10^3^–10^7^ CFU/mL for the seven samples from tissue homogenates included in the present study. Interestingly, the tissue homogenate with low detection in GeneXpert MTB/RIF Ultra presented with the highest genome coverage of the tested samples. The presence of contaminating agents or co-infection with NTM was excluded after targeted amplicon sequencing. The performance of this culture-independent approach on more challenging samples needs to be explored and reported by including samples from microbially-complex upper respiratory tract or sputum specimens.

## Conclusion

By applying a culture-independent approach using NAS on DNA extracted from infected African buffalo tissue samples, we were able to generate high quality *M. bovis* whole-genome sequences. Significant differences were observed in the reference genome position coverage between long- and short-reads, which warrant further investigation. By including comparable genomic positions between sequencing platforms and based on SNP analysis, we confirmed a clonal spread of the pathogen in the investigated buffalo herd. The described approach will be applied to obtain crucial information regarding the epidemiology of animal TB in complex ecosystems at the livestock–wildlife–human interface.

## Data availability statement

The datasets presented in this study can be found in online repositories available at: ncbi.nlm.nih.gov/bioproject/PRJEB63553 under project reference number PRJEB63553.

## Ethics statement

The animal study was approved by Stellenbosch University Animal Care and Use Committee (SU-ACUD15-00072), Stellenbosch University Biological and Environmental Safety Research Ethics Committee (SU-BEE-2021-22561) and Department of Agriculture, Land Reform and Rural Development (ref: 12/11/1/7/6 and 12/11/1/7/2). The study was conducted in accordance with the local legislation and institutional requirements.

## Author contributions

GG: Formal analysis, Investigation, Methodology, Validation, Visualization, Writing – original draft, Writing – review & editing. JL: Data curation, Formal analysis, Writing – review & editing. TK: Data curation, Writing – review & editing. TS: Validation, Writing – review & editing. TT: Data curation, Investigation, Writing – review & editing. LM: Visualization, Writing – review & editing. MO’H: Visualization, Writing – review & editing. SM: Writing – review & editing. AO: Data curation, Writing – review & editing. AL: Funding acquisition, Resources, Writing – review & editing. RW: Conceptualization, Funding acquisition, Resources, Writing – review & editing. MHM: Conceptualization, Investigation, Methodology, Resources, Writing – review & editing. MAM: Conceptualization, Methodology, Resources, Supervision, Writing – review & editing. WG: Conceptualization, Formal analysis, Funding acquisition, Investigation, Methodology, Project administration, Resources, Supervision, Validation, Writing – original draft, Writing – review & editing.

## Footnotes

^1^https://www.bioinformatics.babraham.ac.uk/projects/fastqc

^2^https://github.com/tleonardi/pycoQC

^3^https://github.com/DerrickWood/kraken2

^4^https://github.com/USDA-VS/vSNP3/tree/main

^5^https://github.com/tseemann/snp-dists

^6^https://github.com/raivokolde/pheatmap

^7^https://nsdpy.readthedocs.io/en/latest/main.html#instructions-for-use-from-the-terminal

^8^https://github.com/tseemann/abricate
