# Supplementary material for: Advancing animal tuberculosis surveillance using culture-independent long-read whole-genome sequencing
Source: Front Microbiol. 2023 Nov 21;14:1307440. doi: 10.3389/fmicb.2023.1307440 (PMC10699144; doi:10.3389/fmicb.2023.1307440)
Supplement: Supplementary file 1 [file Data_Sheet_1.zip › Supplementary Material S1.PDF]

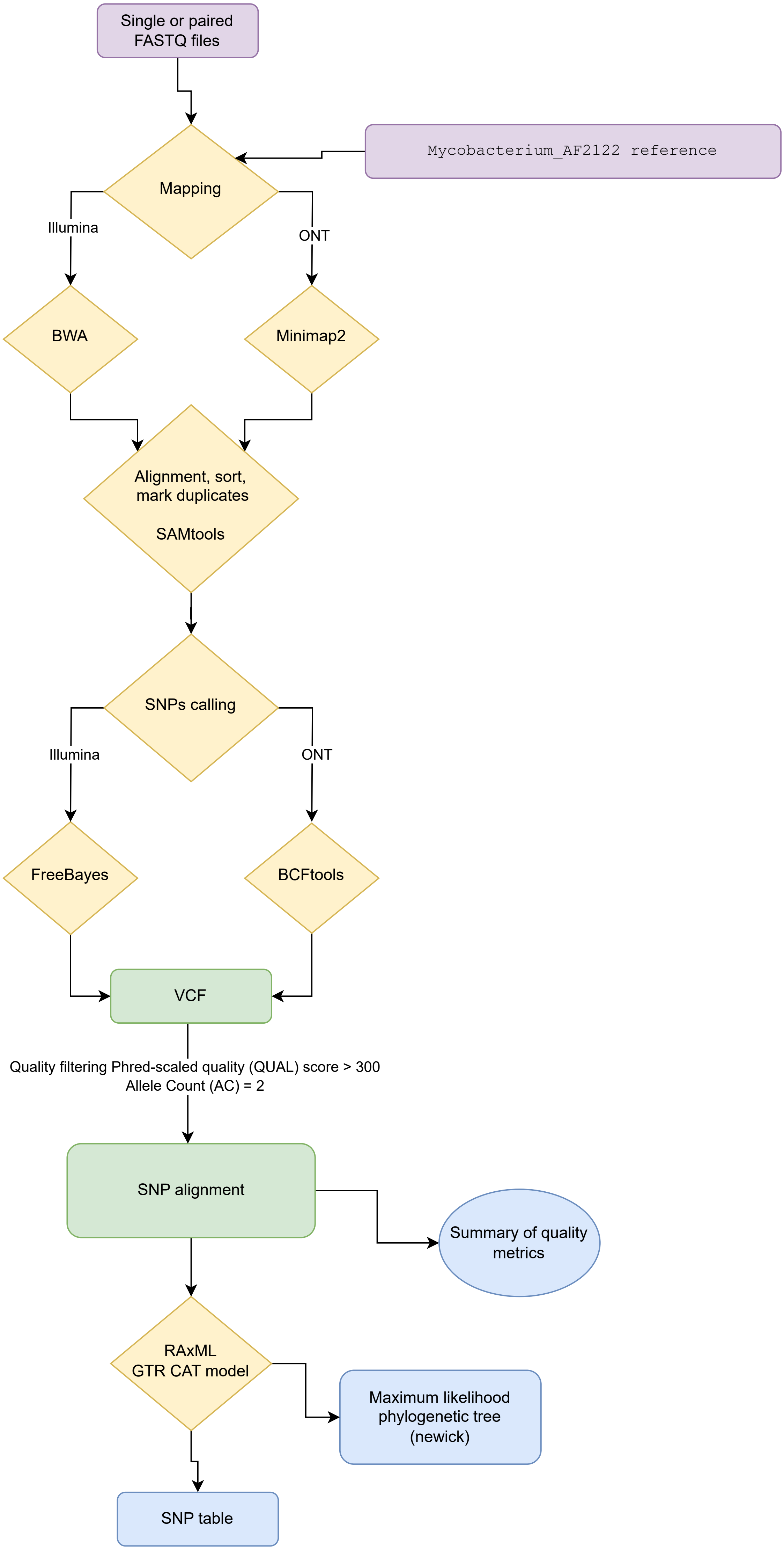

Single or paired FASTQ files
Mycobacterium_AF2122 reference
Mapping
Illumina
ONT
BWA
Minimap2
Alignment, sort, mark duplicates
SAMtools
SNPs calling
Illumina
ONT
FreeBayes
BCFtools
VCF
Quality filtering Phred-scaled quality (QUAL) score > 300
Allele Count (AC) = 2
SNP alignment
Summary of quality metrics
RAxML
GTR CAT model
Maximum likelihood phylogenetic tree (newick)
SNP table

The vSNP3 pipeline involves a two-step process[1], including mapping of genomic short- and long-reads and variant calling based on predefined quality filters. In Step 1, mapped genomic short- and long-reads using the Burrows-Wheeler Aligner (BWA)[1] and Minimap2 (v2.26)[2] against the reference genome *M. bovis* AF2122/97 (Accession NC_002945.4). Thereafter, SAMtools (v1.17) was used to fix mate information, sort alignments, and mark duplicates[3]. Finally, SNP positions were called using FreeBayes (v1.3.6 https://github.com/freebayes/freebayes), a haplotype-based variant detector, and BCFtools (v1.17)[3], generating variant call format files from short- and long-read derived alignments, respectively. Results were filtered using a minimum Phred-scaled quality (QUAL) score of 150 and an Allele Count (AC) of 2. SNP position (AC = 1 and present in < 90% of the reads) was considered ambiguous as coded by the International Union of Pure and Applied Chemistry (IUAPC) and based on visual inspection. Nanopore QUAL values were increased by 100 to more similar values seen with Illumina reads, which allowed VCF files from both platforms to be run together. SNP positions that had a variant call in more than 90% of reads were considered homozygous whereas variant calls identified in < 90% of the reads were considered heterozygous and removed from the analysis. Defining SNPs were used to identify different groups of isolates, as specified in the dependency files of the vSNP3 pipeline. A summary of quality metrics was then generated to evaluate the performance of the sequencing run of each isolate. This included the average depth of coverage, the average read length, the percent of the reference genome covered by the reads from each isolate, the number of contigs not mapping to the reference, the number of SNPs with a QUAL score of >150 for Illumina and >250 for ONT and with an AC of 2 (good SNPs), and the spoligotype octal code. The octal code was based on the counts of each spacer sequence against the raw FASTQ files.

In Step 2, after performing various filtering steps to remove error-prone regions variant positions, informative SNPs were used to create SNPs tables and phylogenetic trees. The filtering process involved excluding SNPs in repetitive regions using default masking files in the vSNP3 dependencies to account for highly GC-rich and polymorphic proline-glutamate (PE)/proline-proline-glutamate (PPE) gene family, for this purpose, based on previous observations[4]. Additionally, SNPs in areas with an anomalous accumulation of variants were omitted. Thereafter, SNPs called between related isolate groups were assessed based on specific criteria: at least one locus with an allele count of 2, a quality score greater than 150, and map quality greater than 56, following standard thresholds in vSNP3.

In conjunction with the vSNP3 pipeline, MINTyper[5], designed to compare sequences from both the Illlumina and ONT platform, were implemented to determine genetic distance between the sequences and estimate a distance matrix. Briefly, reads were aligned to the AF212297 (Accession NC_002945.4) reference genome using KMA v1.3.8, employing preset options "-mint2" for Illumina and "-mint3" for ONT sequences. KMA generated consensus sequences with uppercase bases for significant positions and lowercase for non-significant ones. SNP-calling criteria for "-mint2" included unambiguous mapping, ≥ 10X depth, ≥ 90% support, and significant overrepresentation (McNemar test, α = 0.05); "-mint3" used ≥ 10X depth, ≥ 70% support, and significant overrepresentation. Alignments were trimmed using CCPhylo v0.2.2, retaining SNP-calling compliant positions, removing adjacent SNPs within 10 bases, and eliminating CCWGG motifs from ONT data. Hierarchical clustering employed CCPhylo's Neighbor-Joining, and phylogenetic trees were generated using IQtree v2.0.3 (parameters: "-seqtype DNA -seed 256")[6] and FastTree v2.1.11 (parameters: "-gtr –nt")[7].

**References**

1. Li, H. Aligning sequence reads, clone sequences and assembly contigs with BWA-MEM. 0 Bytes (2014).
2. Li, H. Minimap2: pairwise alignment for nucleotide sequences. *Bioinformatics* **34**, 3094–3100 (2018).

3. Danecek, P. *et al.* Twelve years of SAMtools and BCFtools. *GigaScience* **10**, giab008 (2021).
4. Cole, S. T. *et al.* Deciphering the biology of *Mycobacterium tuberculosis* from the complete genome sequence. *Nature* **393**, 537–544 (1998).
5. Hallgren, M. B., Overballe-Petersen, S., Lund, O., Hasman, H. & Clausen, P. T. L. C. MINTyper: an outbreak-detection method for accurate and rapid SNP typing of clonal clusters with noisy long reads. *Biol. Methods Protoc.* **6**, bpab008 (2021).
6. Minh, B. Q. *et al.* IQ-TREE 2: New models and efficient methods for phylogenetic inference in the genomic era. *Mol. Biol. Evol.* **37**, 1530–1534 (2020).
7. Price, M. N., Dehal, P. S. & Arkin, A. P. FastTree 2-approximately maximum-likelihood trees for large alignments. *PLoS One* **5**, e9490 (2010).
