# Supplementary material for: Advancing animal tuberculosis surveillance using culture-independent long-read whole-genome sequencing
Source: Front Microbiol. 2023 Nov 21;14:1307440. doi: 10.3389/fmicb.2023.1307440 (PMC10699144; doi:10.3389/fmicb.2023.1307440)
Supplement: Supplementary file 1 [file Data_Sheet_1.zip › Supplementary Material S4.PPTX]

## Slide 1
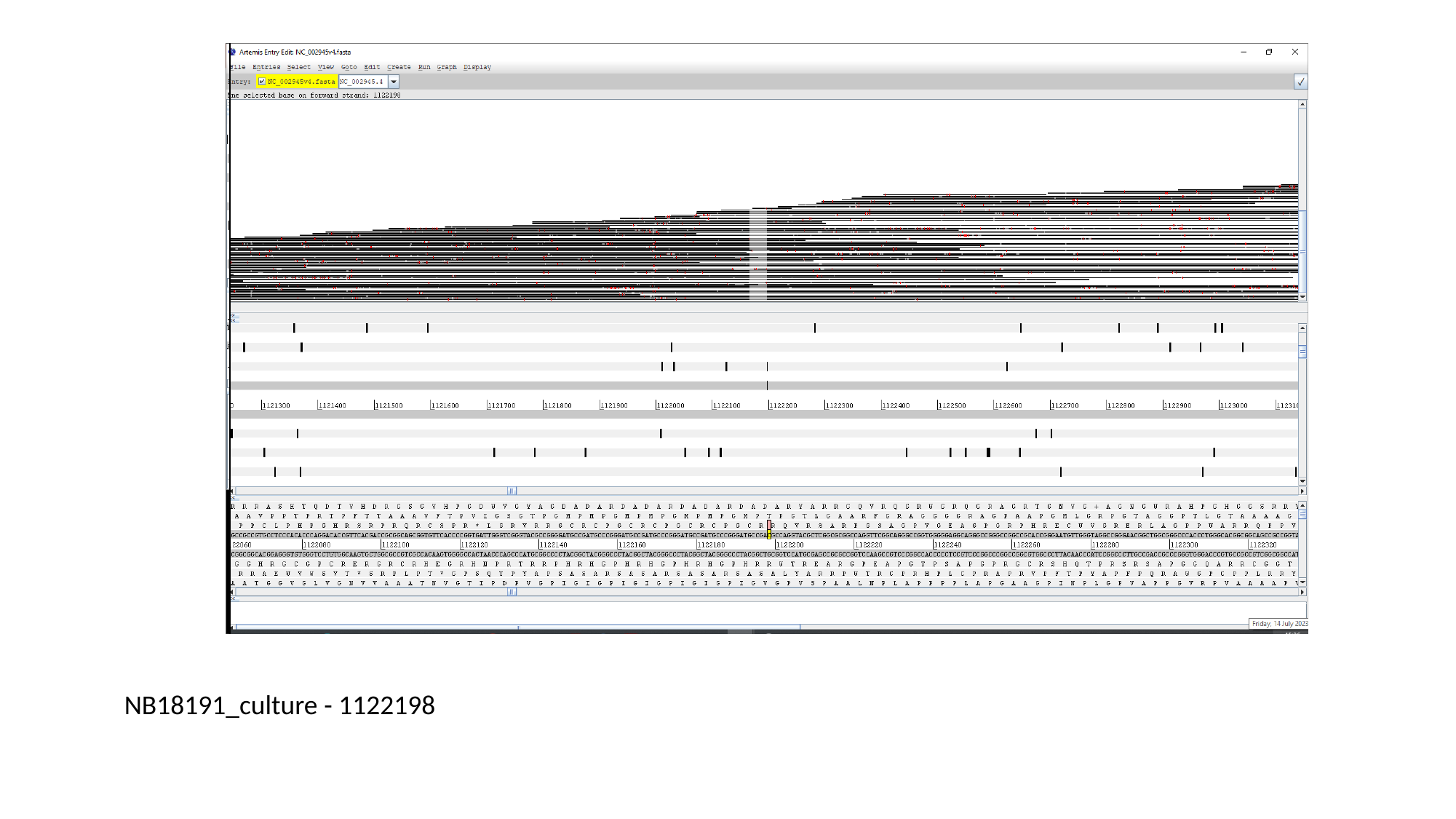

NB18191_culture - 1122198

## Slide 2
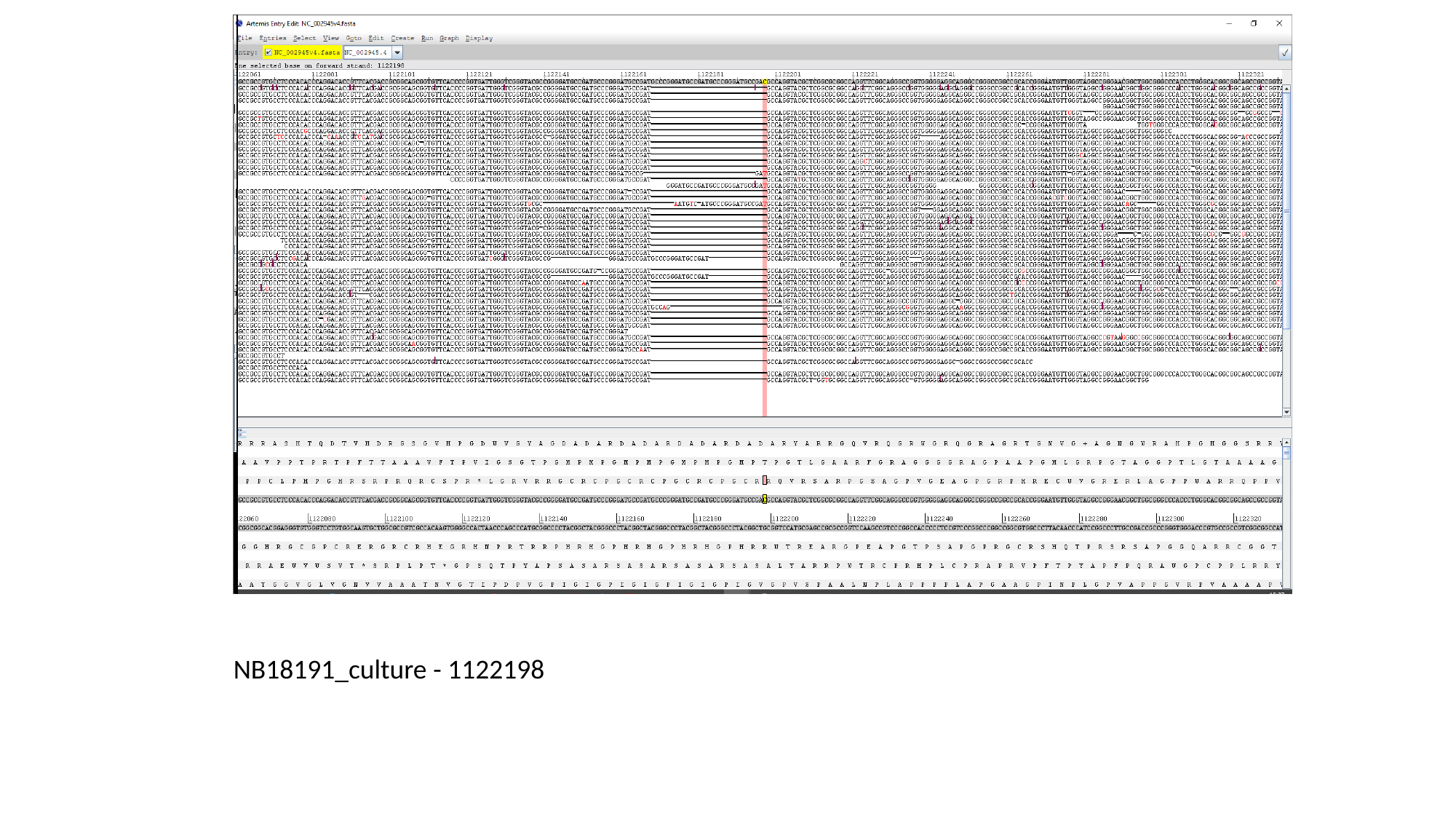

NB18191_culture - 1122198

## Slide 3
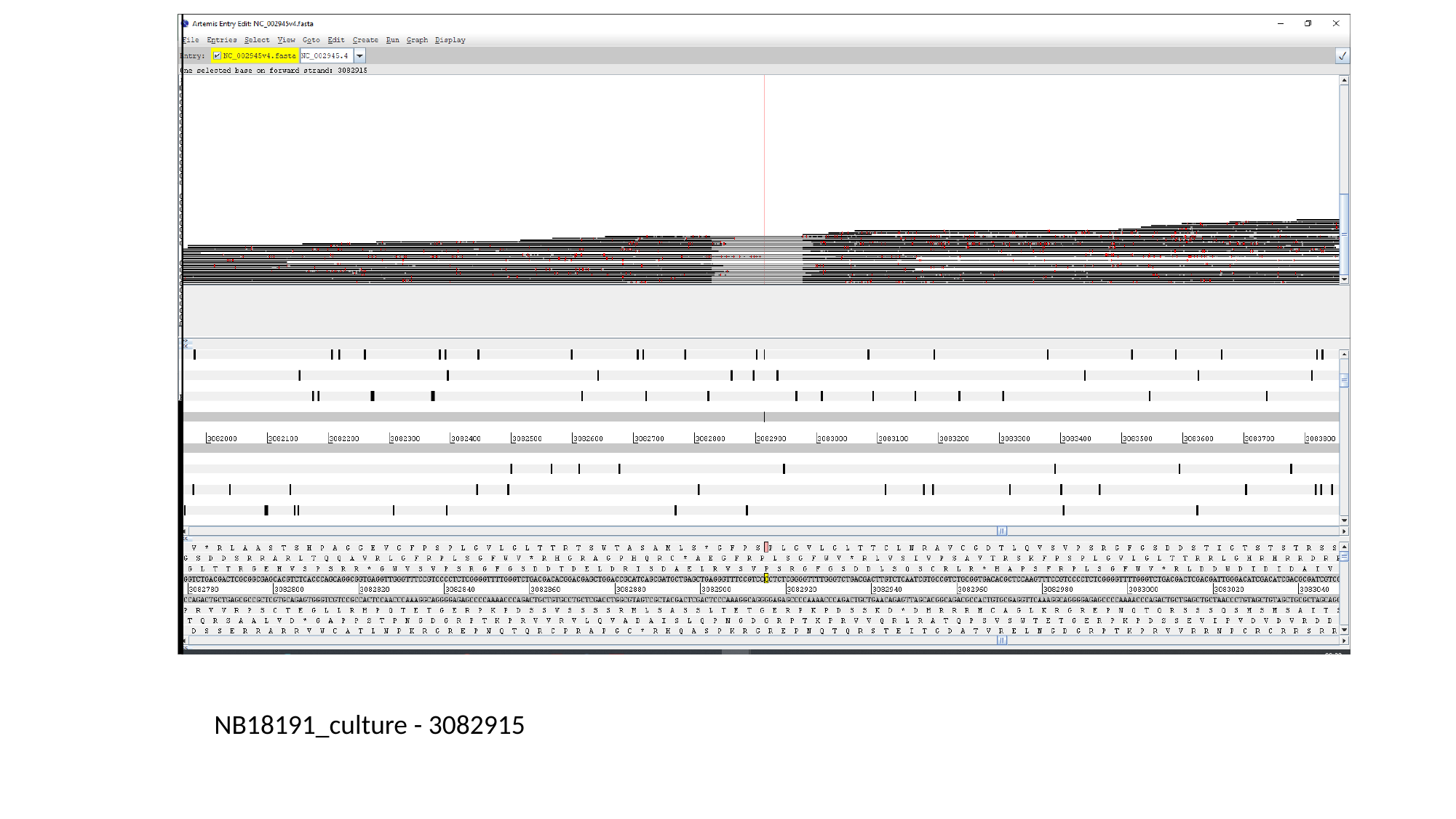

NB18191_culture - 3082915

## Slide 4
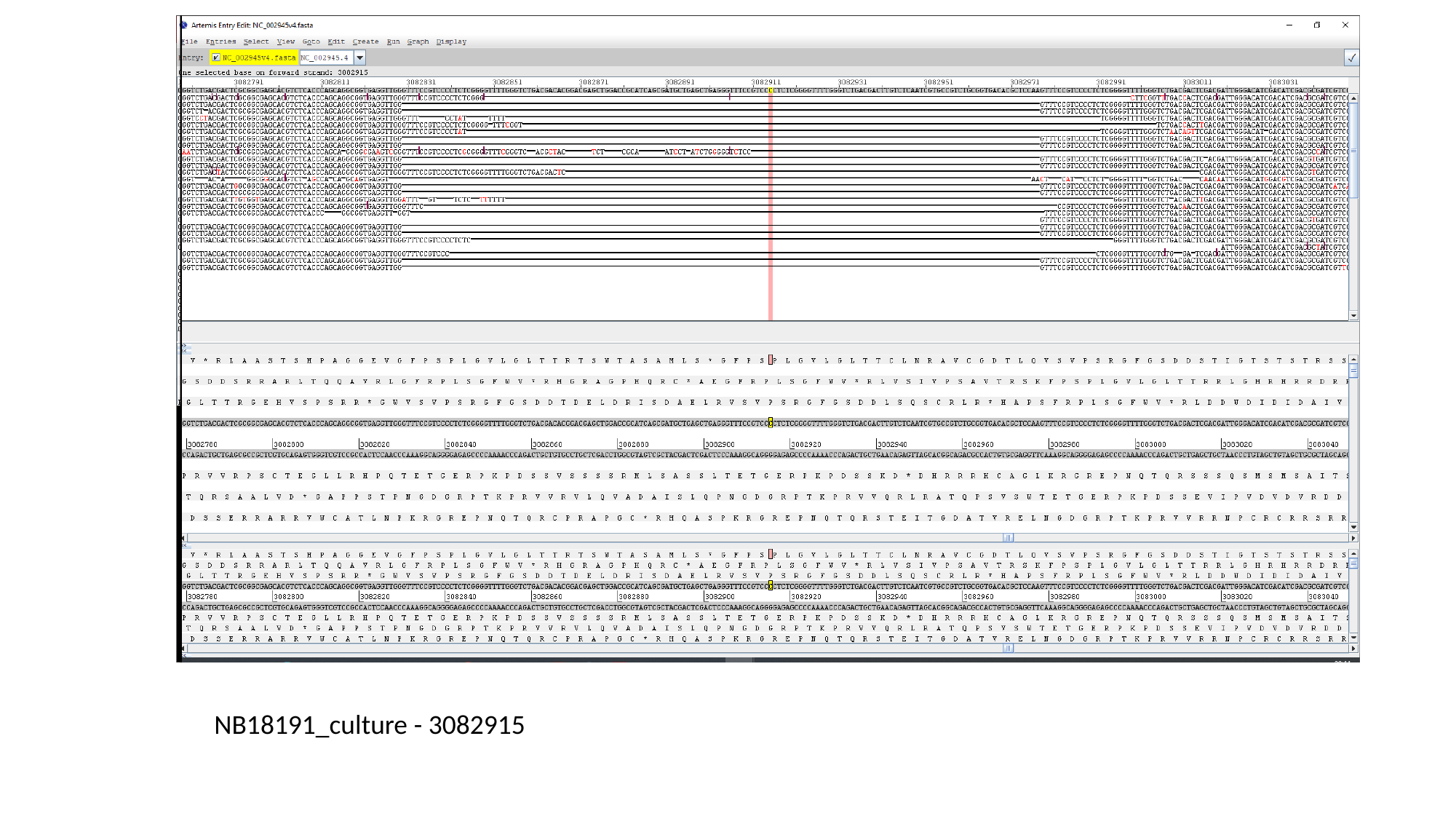

NB18191_culture - 3082915

## Slide 5
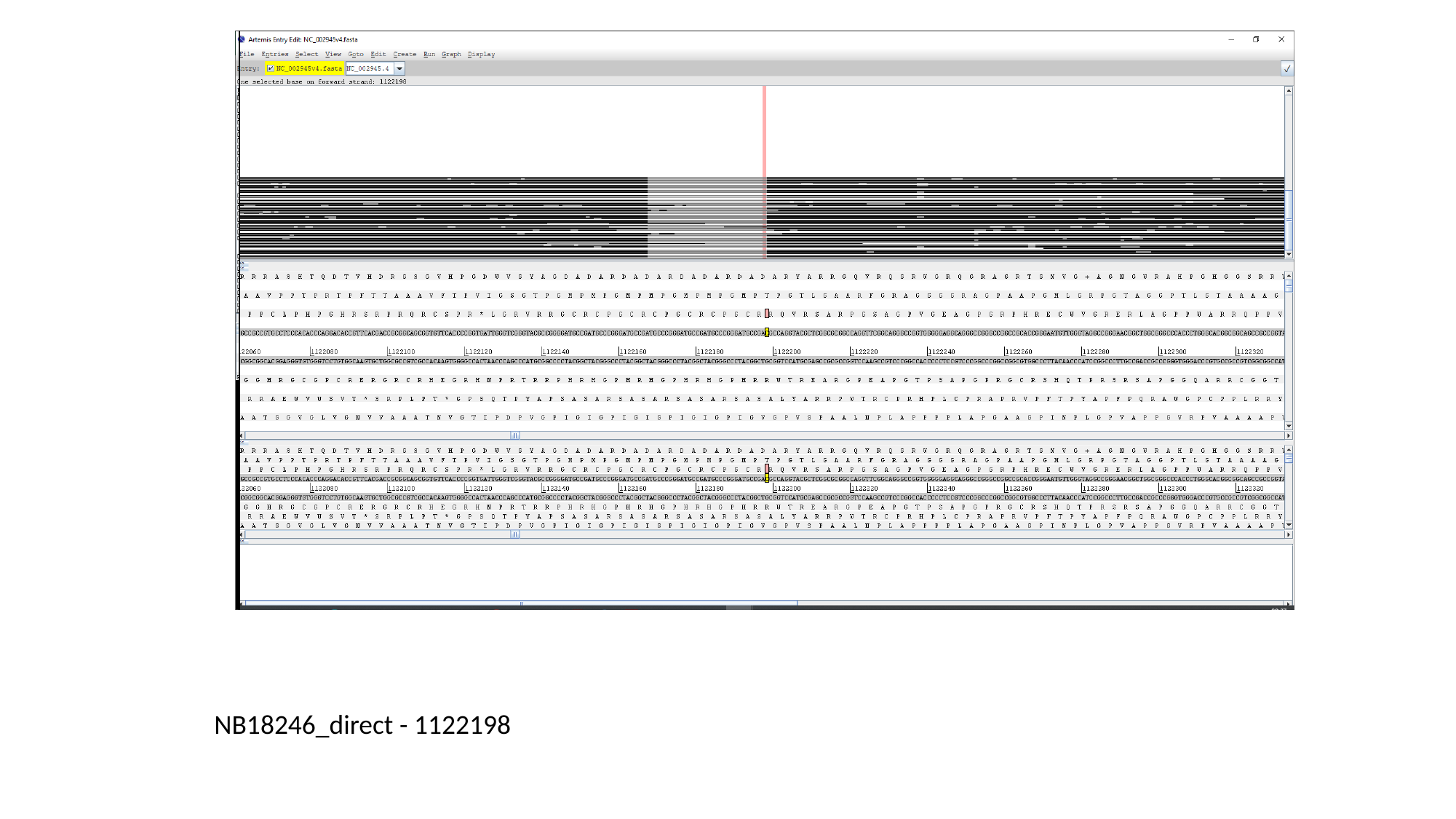

NB18246_direct - 1122198

## Slide 6
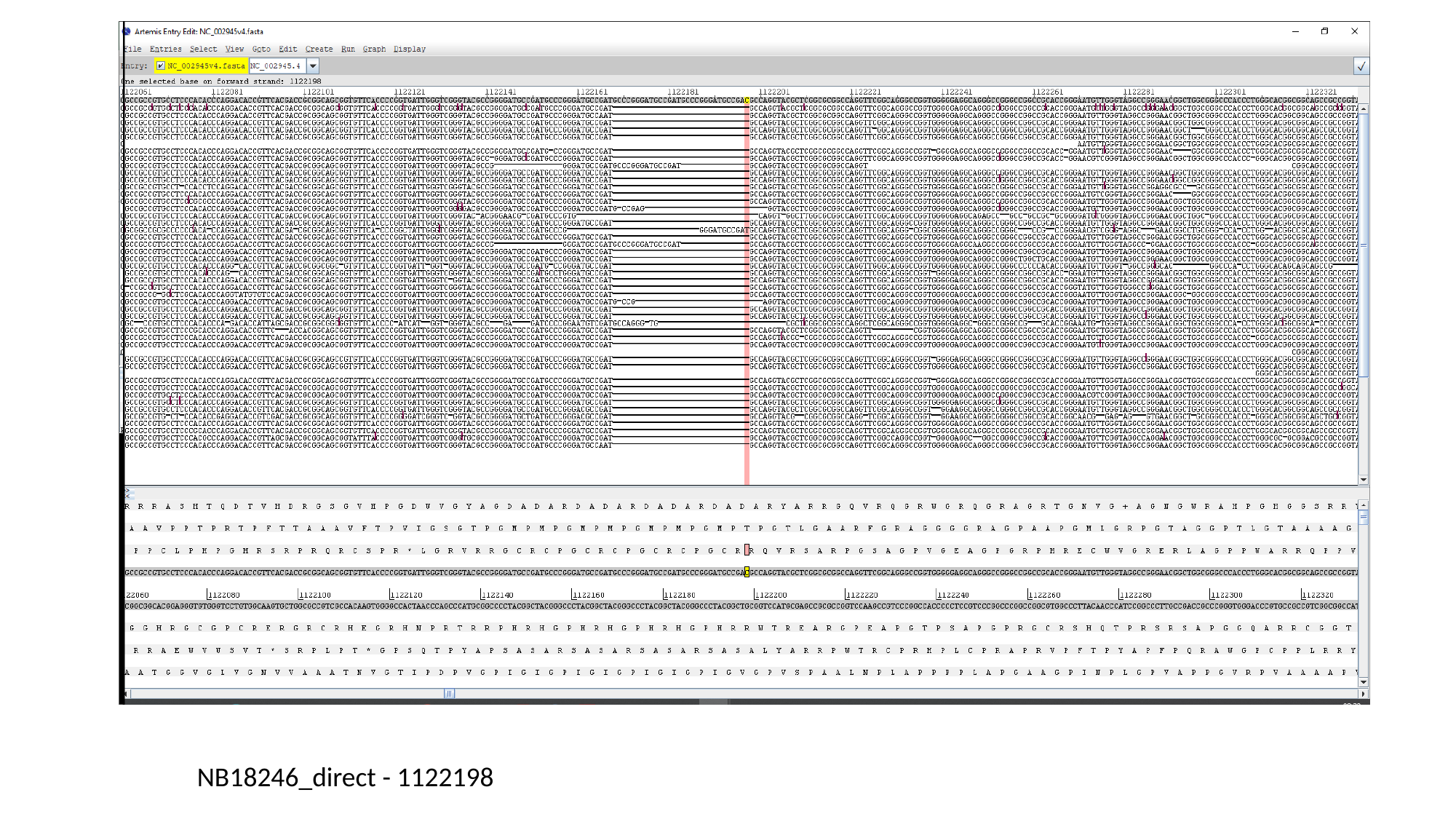

NB18246_direct - 1122198

## Slide 7
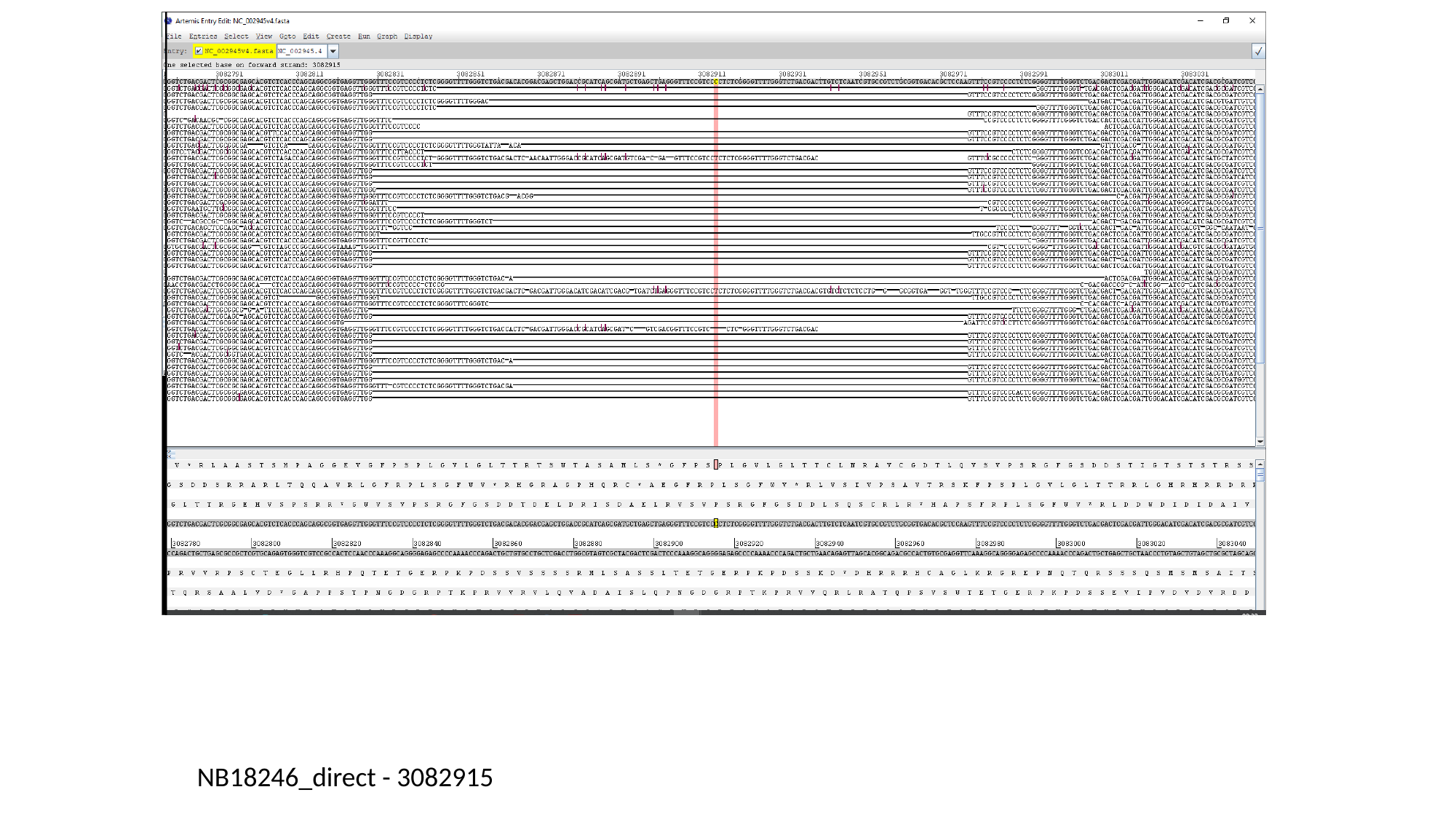

NB18246_direct - 3082915

## Slide 8
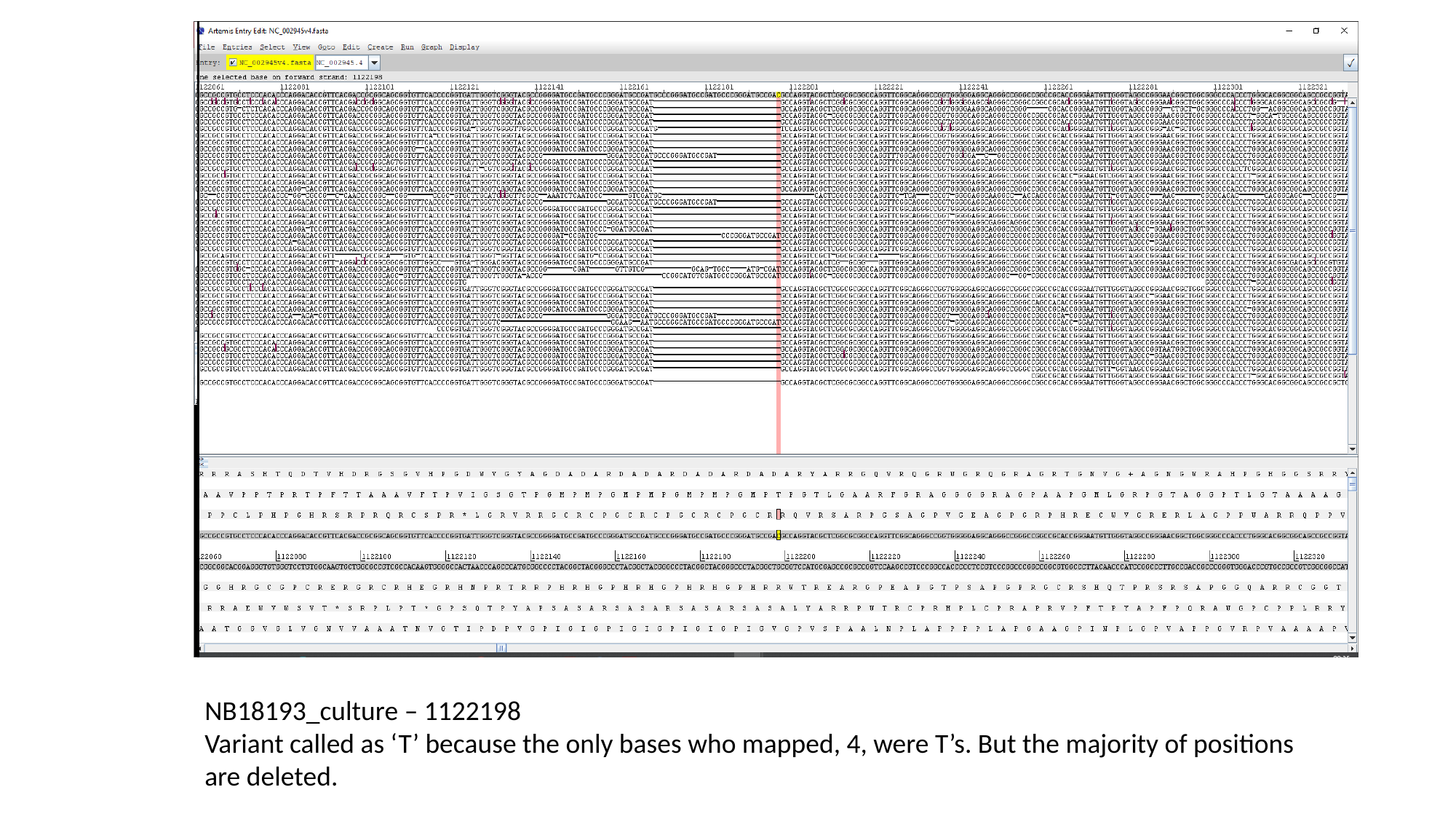

NB18193_culture – 1122198
Variant called as ‘T’ because the only bases who mapped, 4, were T’s. But the majority of positions are deleted.

## Slide 9
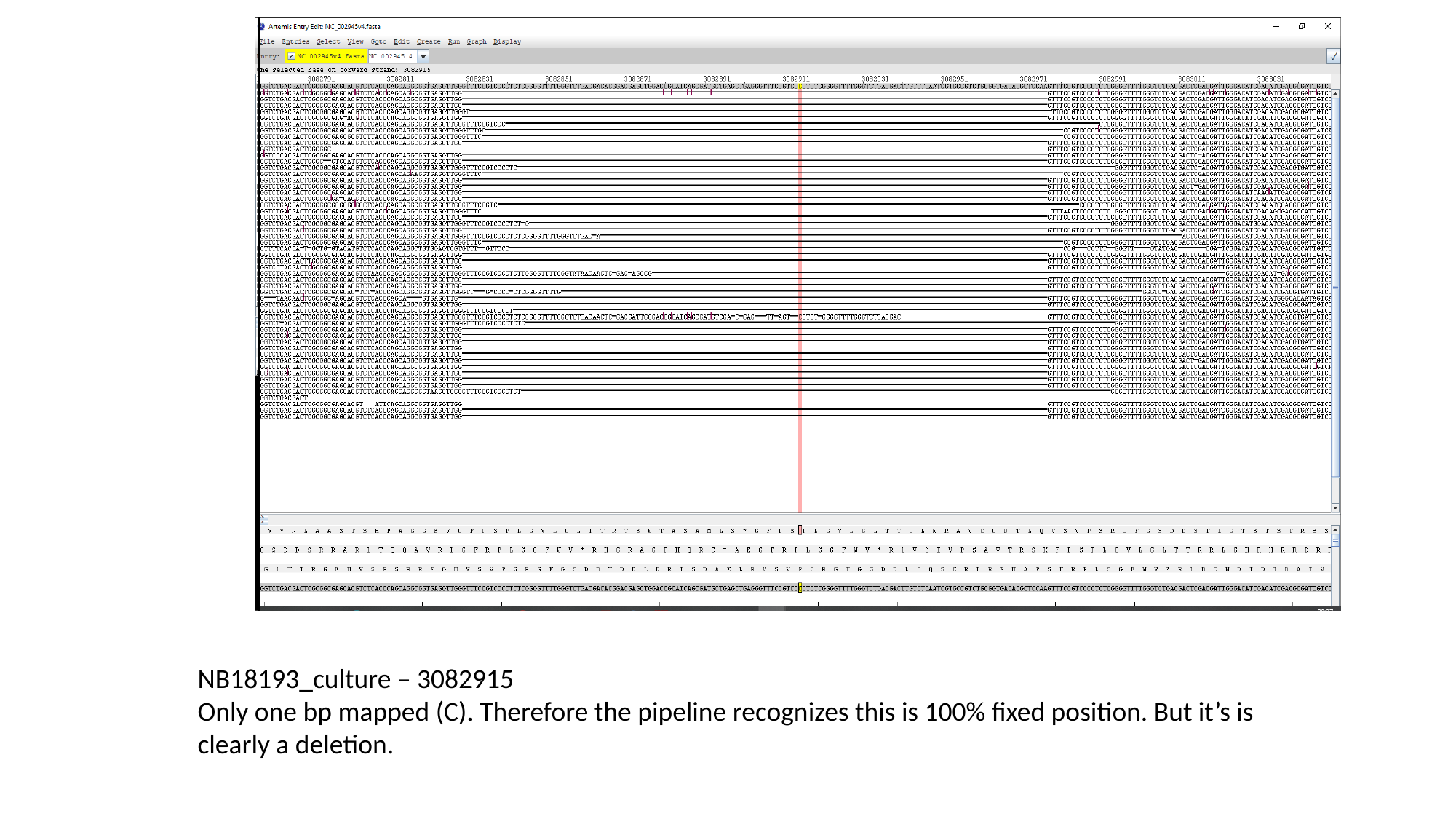

NB18193_culture – 3082915
Only one bp mapped (C). Therefore the pipeline recognizes this is 100% fixed position. But it’s is clearly a deletion.

## Slide 10
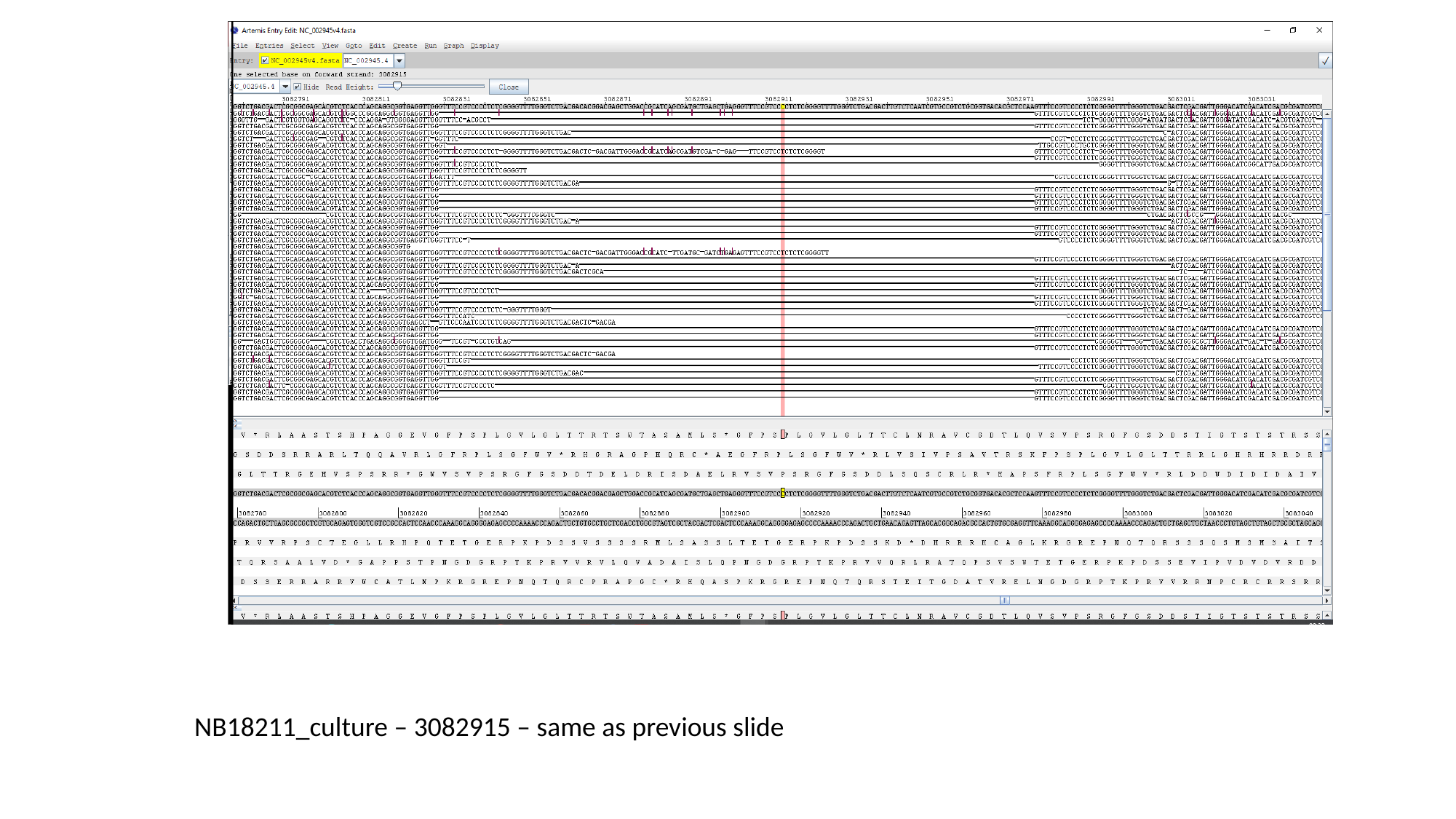

NB18211_culture – 3082915 – same as previous slide

## Slide 11
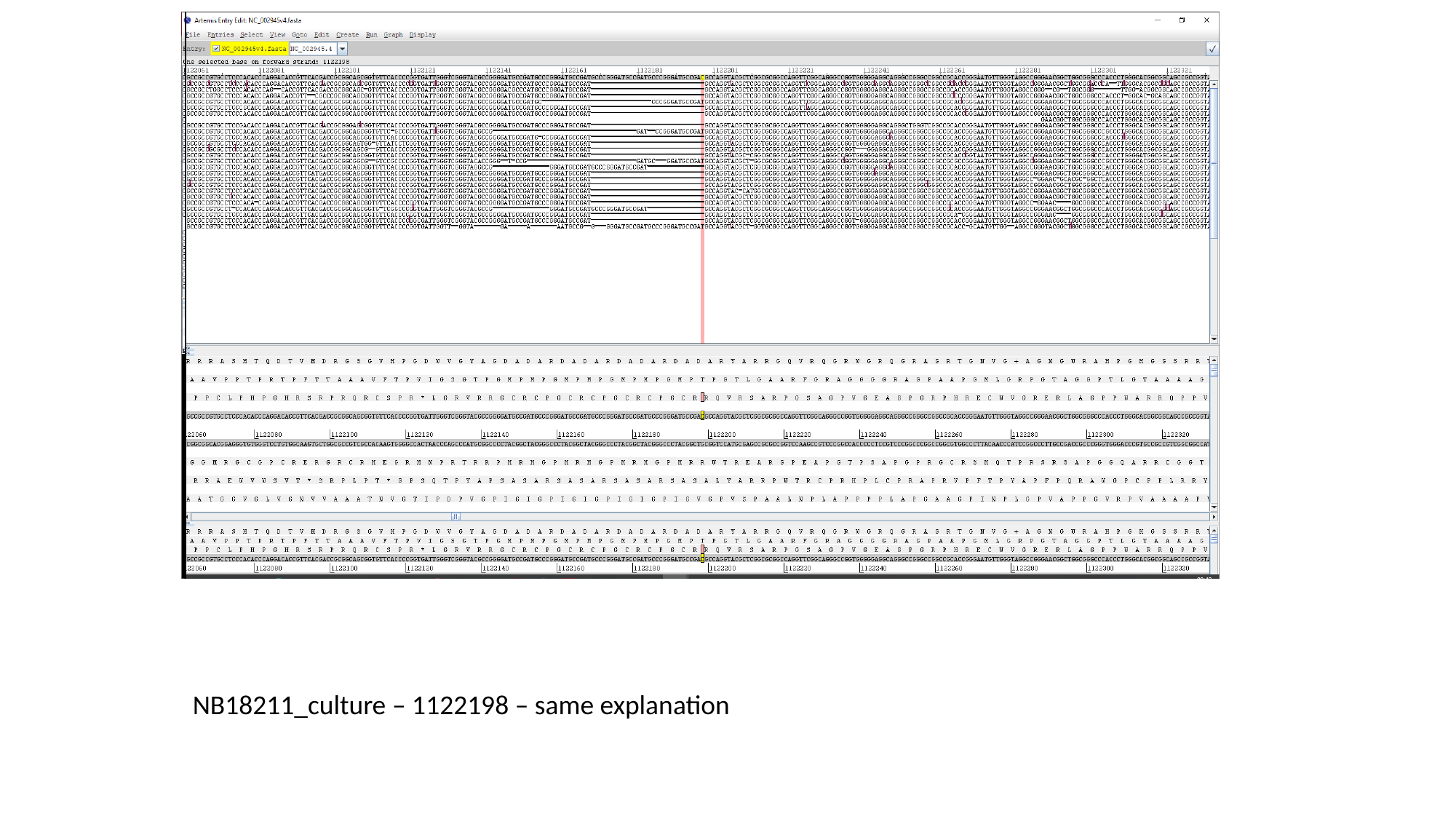

NB18211_culture – 1122198 – same explanation

## Slide 12
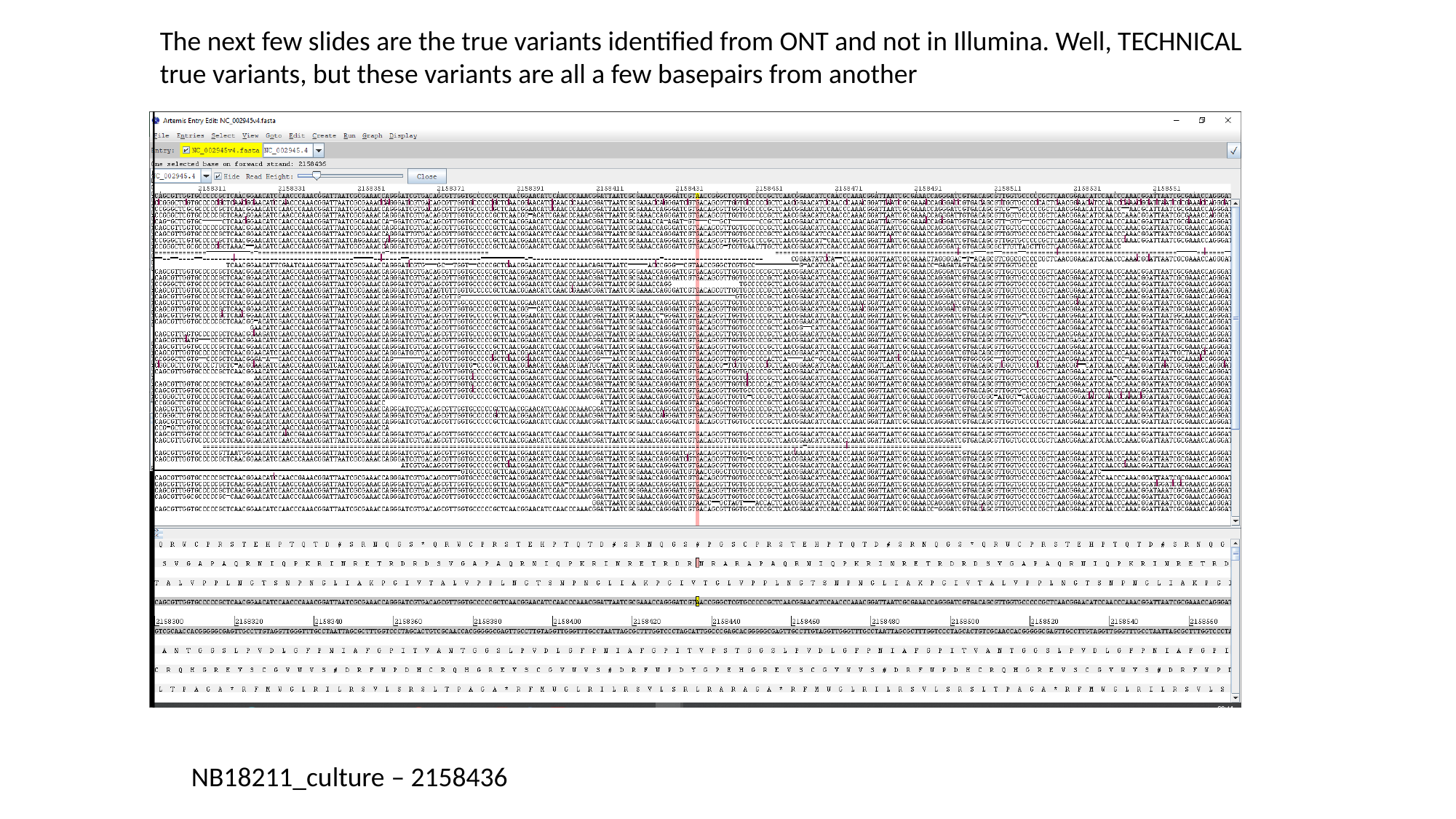

The next few slides are the true variants identified from ONT and not in Illumina. Well, TECHNICAL true variants, but these variants are all a few basepairs from another
NB18211_culture – 2158436

## Slide 13
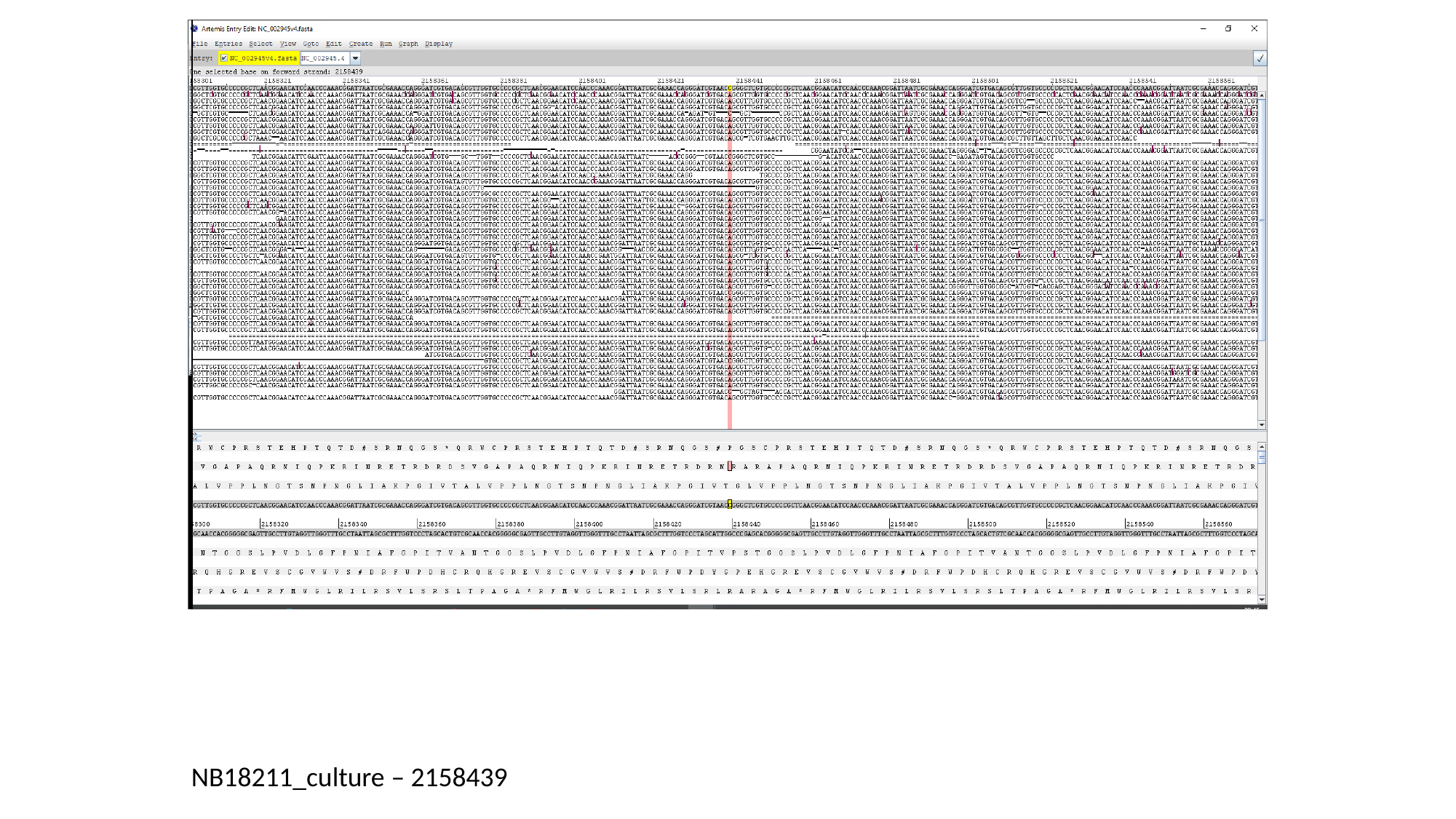

NB18211_culture – 2158439

## Slide 14
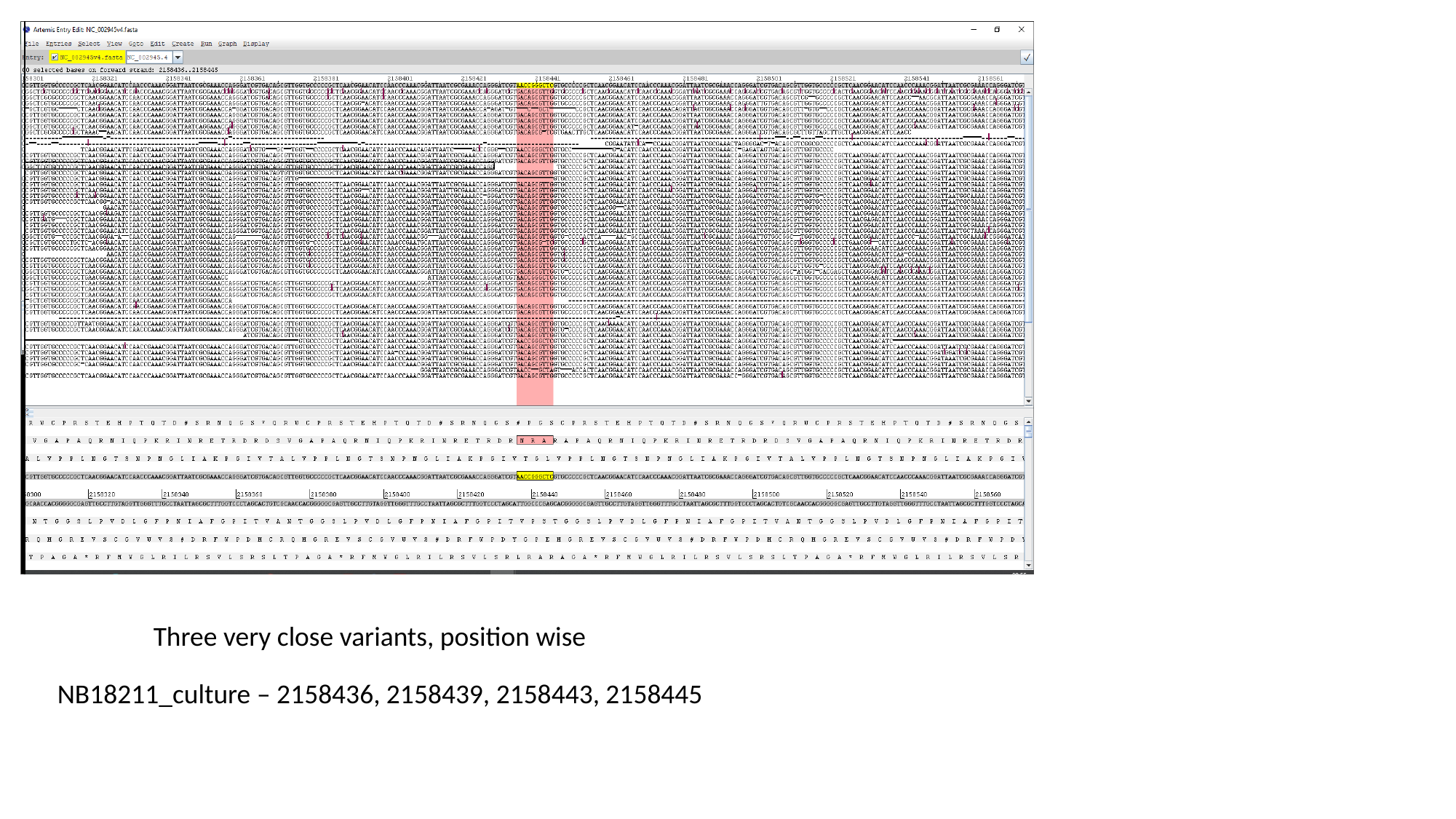

Three very close variants, position wise
NB18211_culture – 2158436, 2158439, 2158443, 2158445

## Slide 15
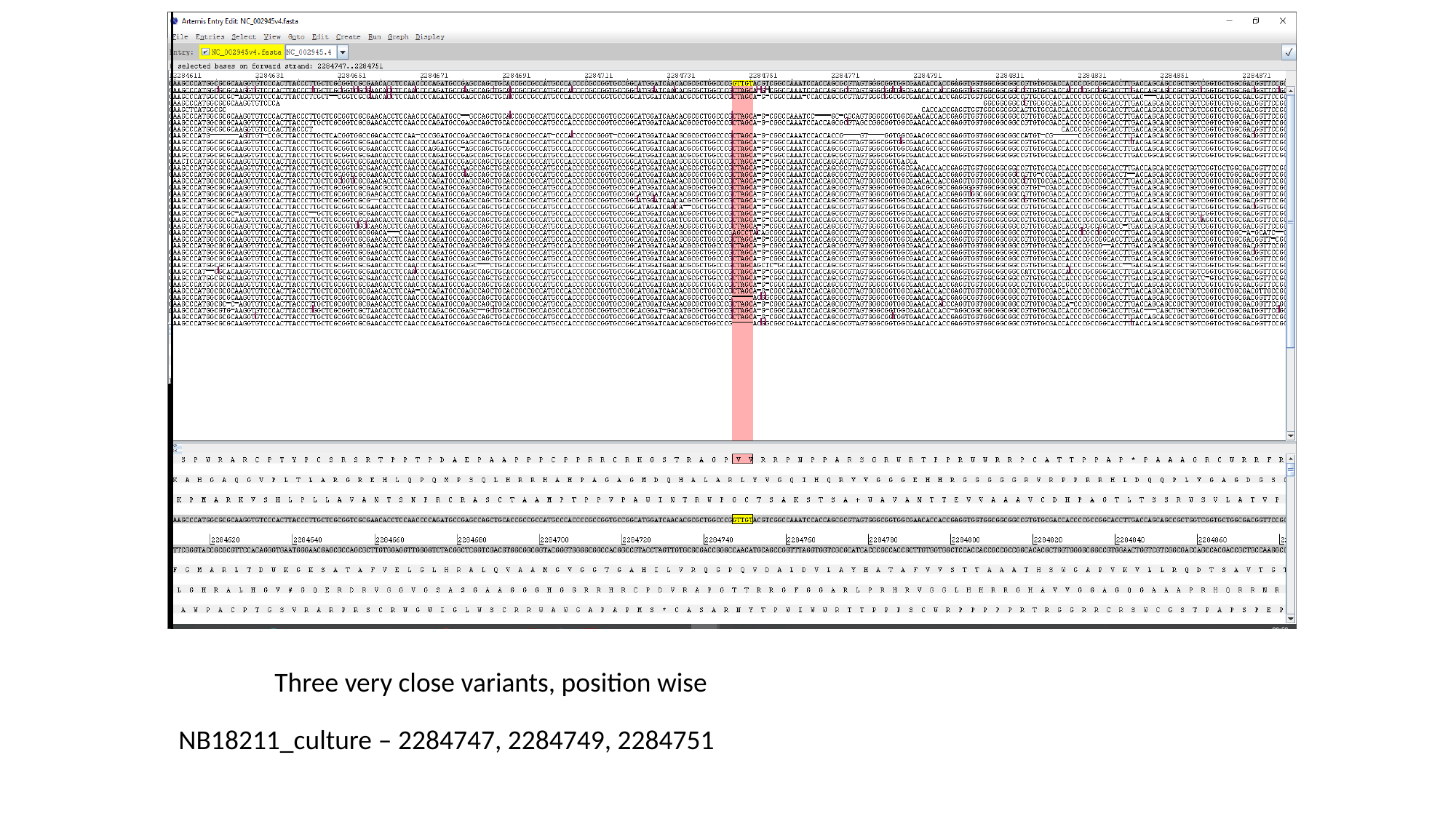

Three very close variants, position wise
NB18211_culture – 2284747, 2284749, 2284751

## Slide 16
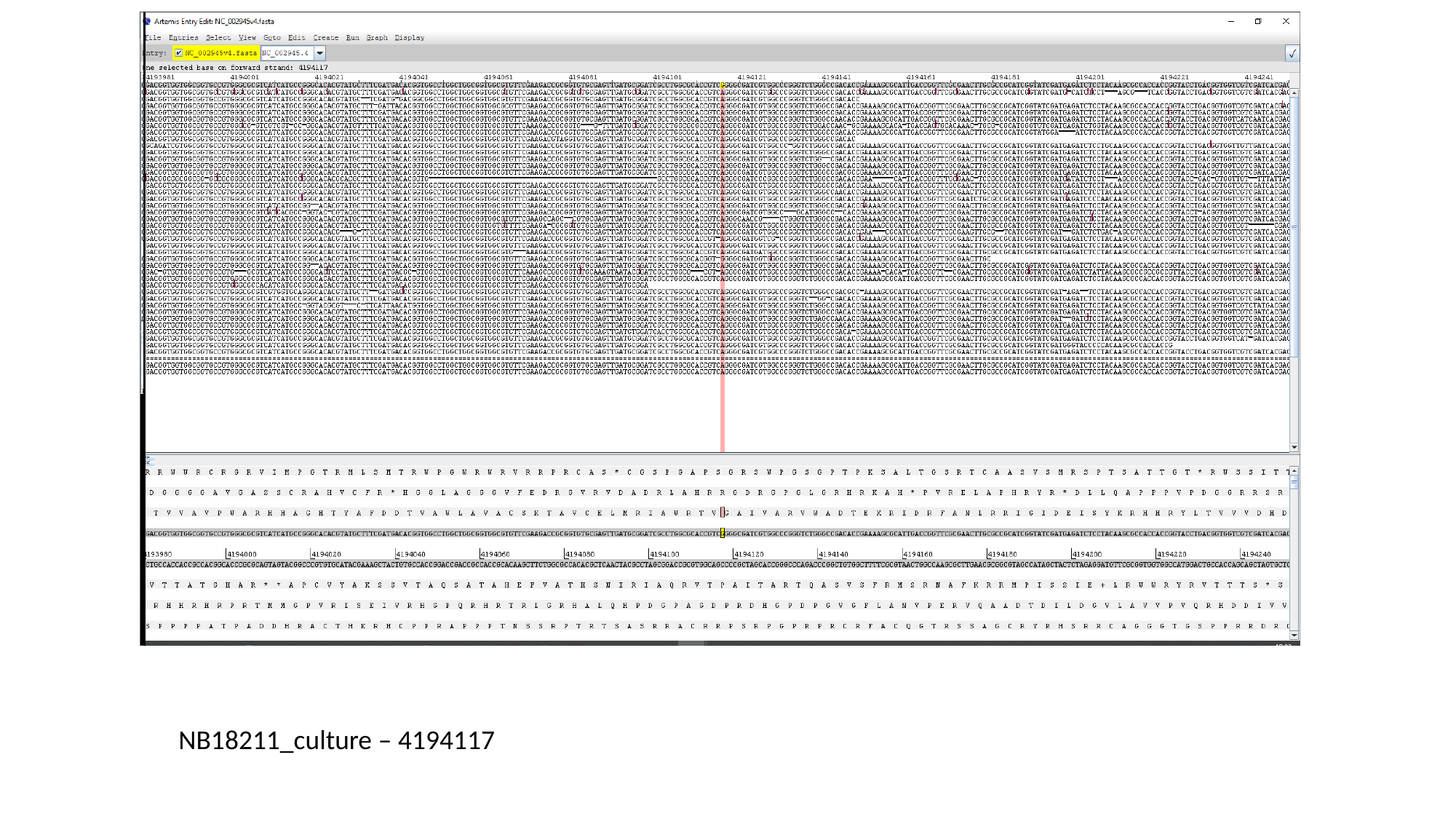

NB18211_culture – 4194117

## Slide 17
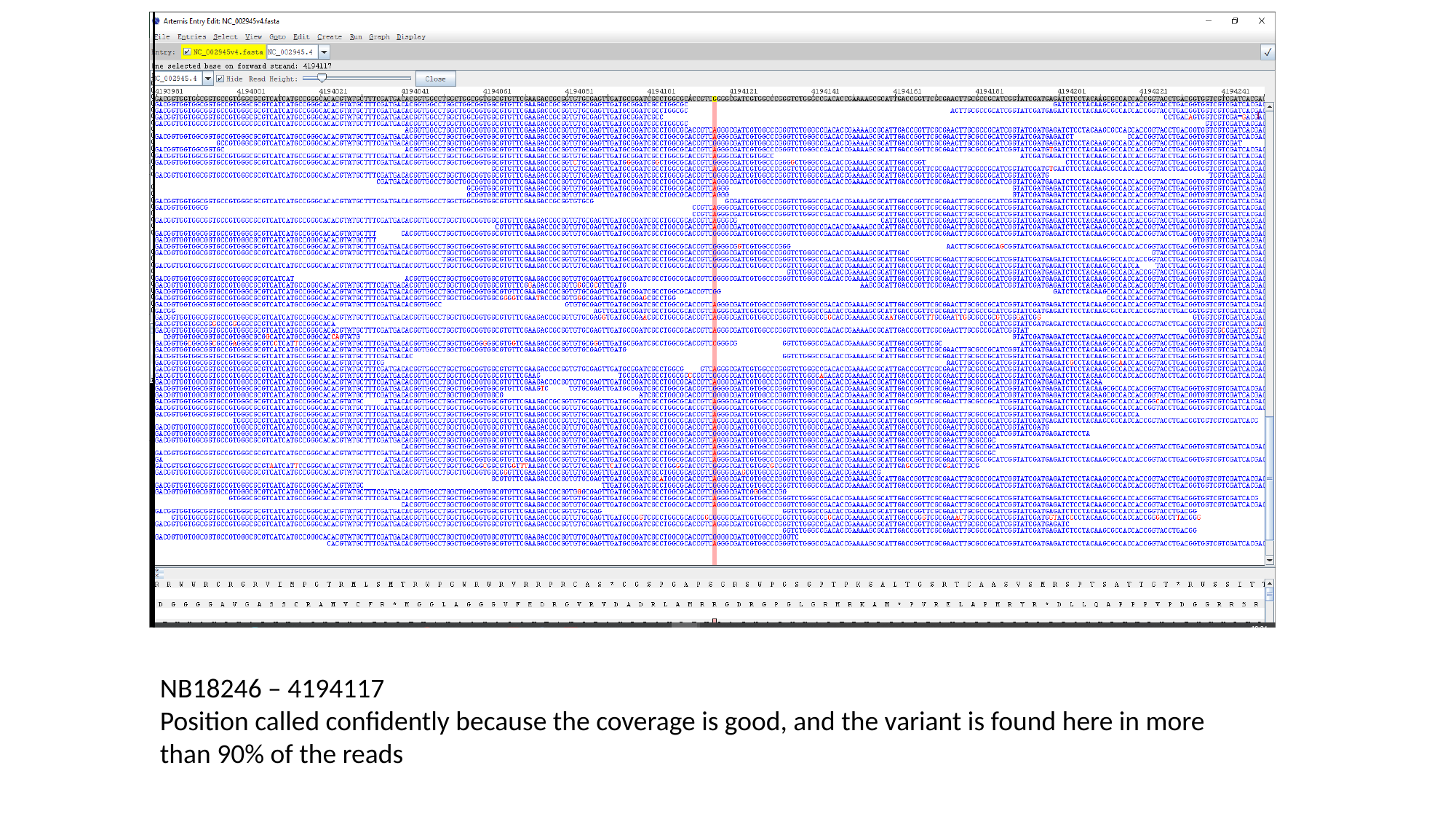

NB18246 – 4194117
Position called confidently because the coverage is good, and the variant is found here in more than 90% of the reads

## Slide 18
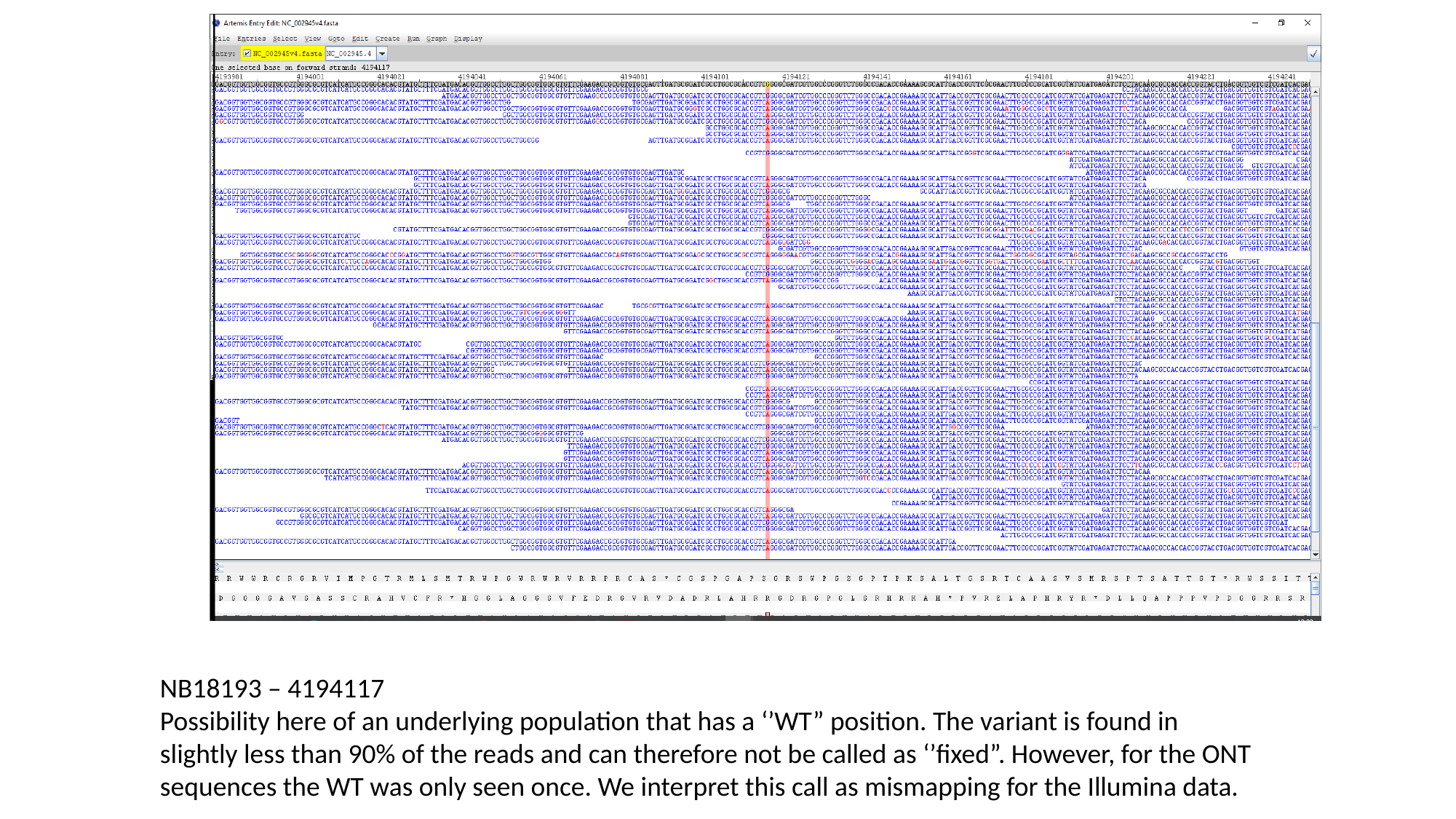

NB18193 – 4194117
Possibility here of an underlying population that has a ‘’WT” position. The variant is found in slightly less than 90% of the reads and can therefore not be called as ‘’fixed”. However, for the ONT sequences the WT was only seen once. We interpret this call as mismapping for the Illumina data.
