# Supplementary figures and images for: Advancing animal tuberculosis surveillance using culture-independent long-read whole-genome sequencing
Source: Front Microbiol. 2023 Nov 21;14:1307440. doi: 10.3389/fmicb.2023.1307440 (PMC10699144; doi:10.3389/fmicb.2023.1307440)

**
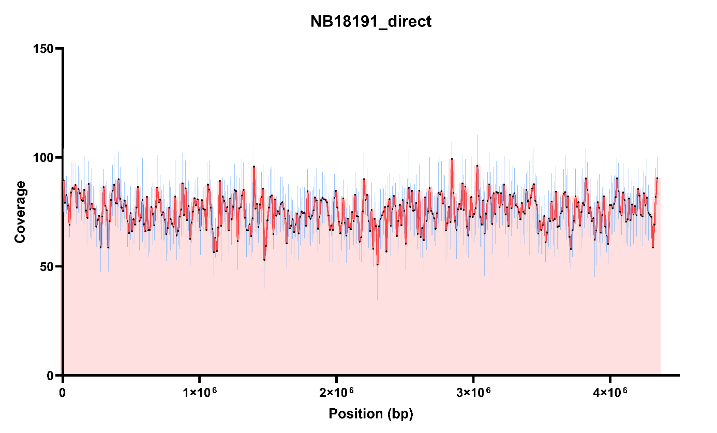

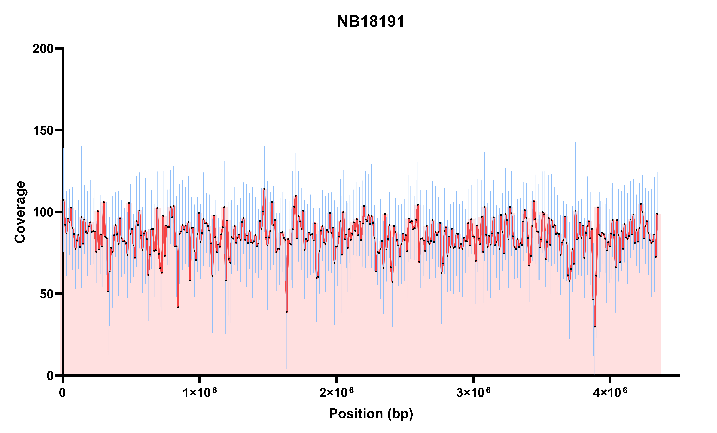
Coverage across the reference.**


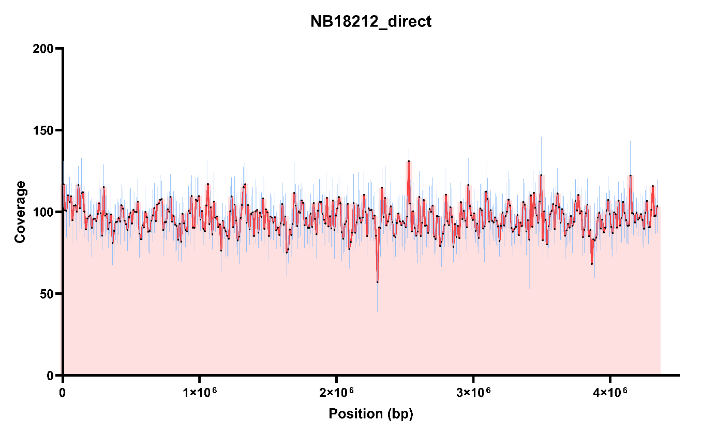

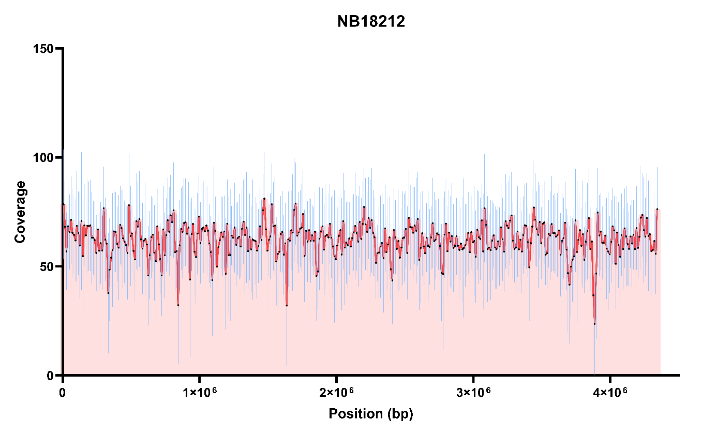


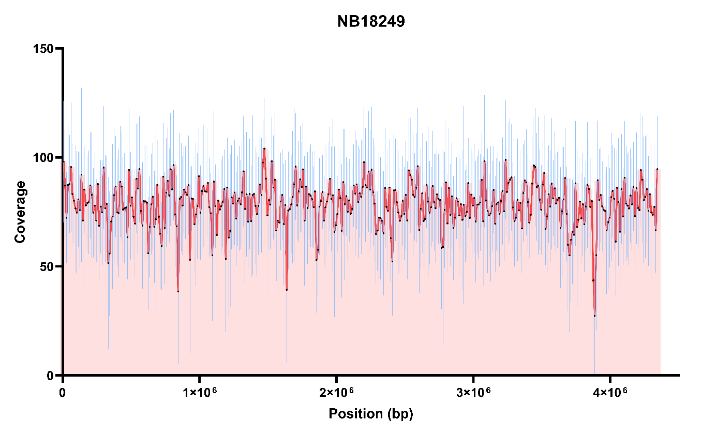

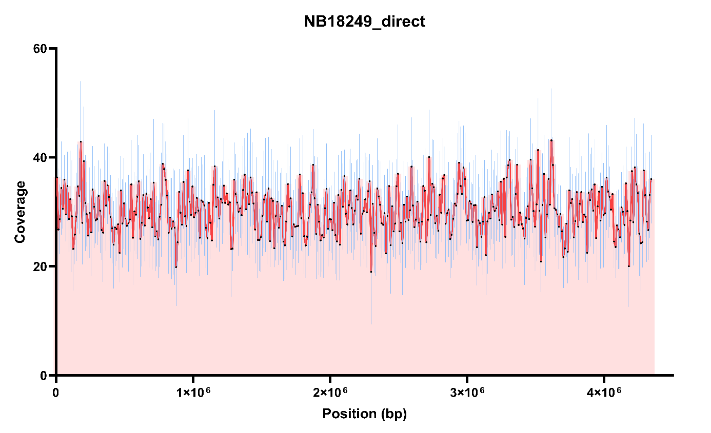


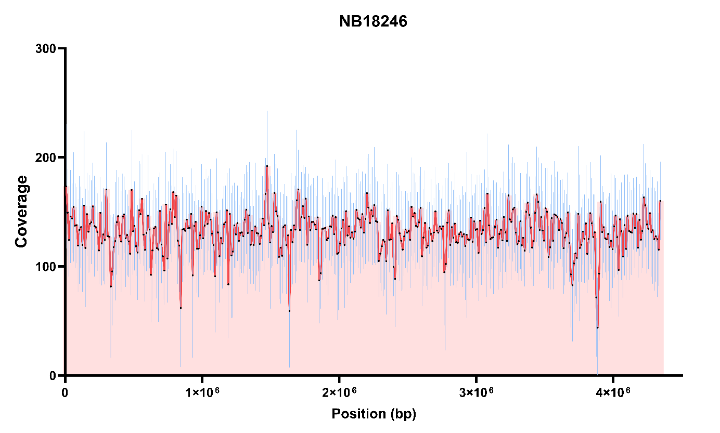

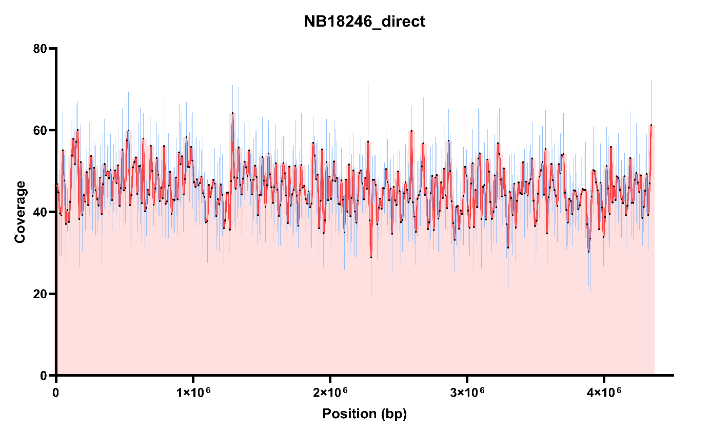


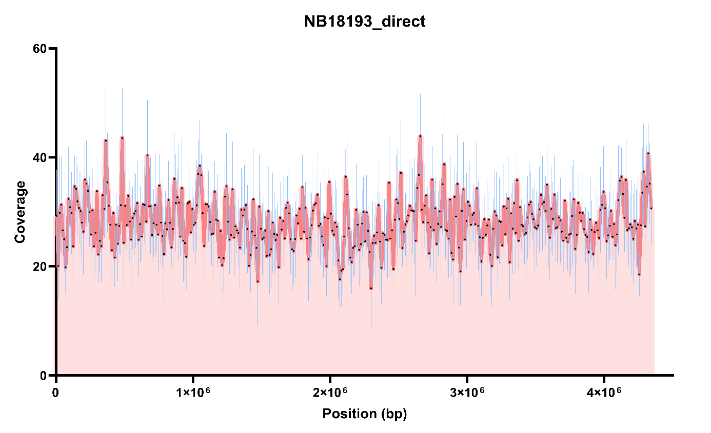

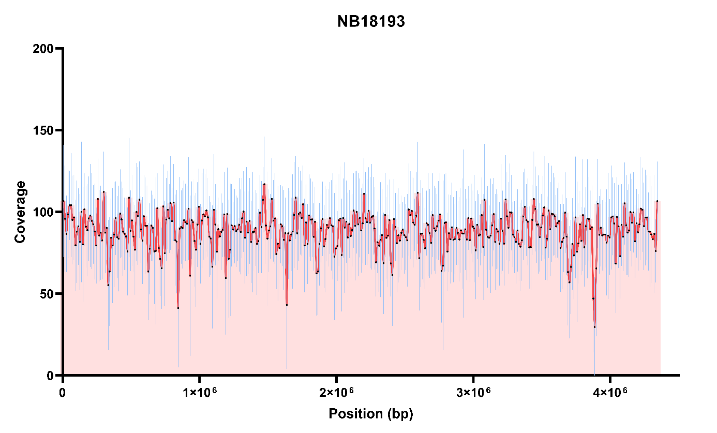


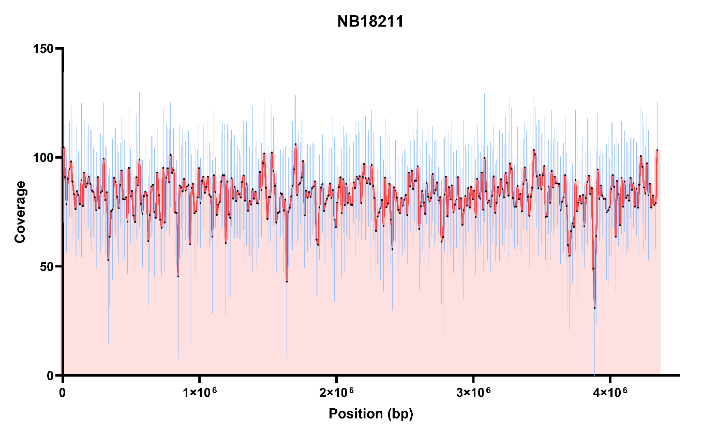

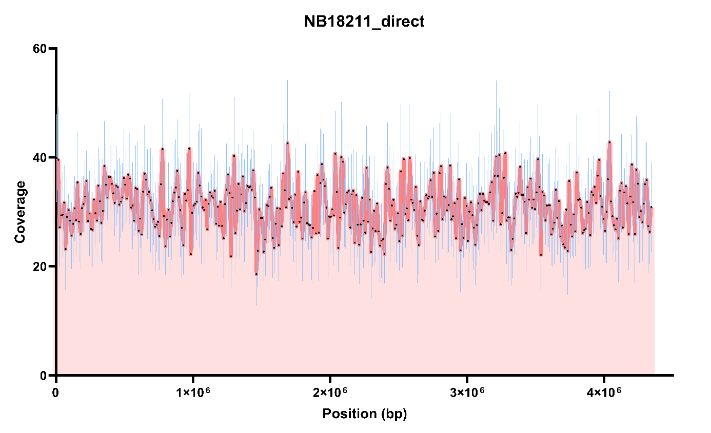


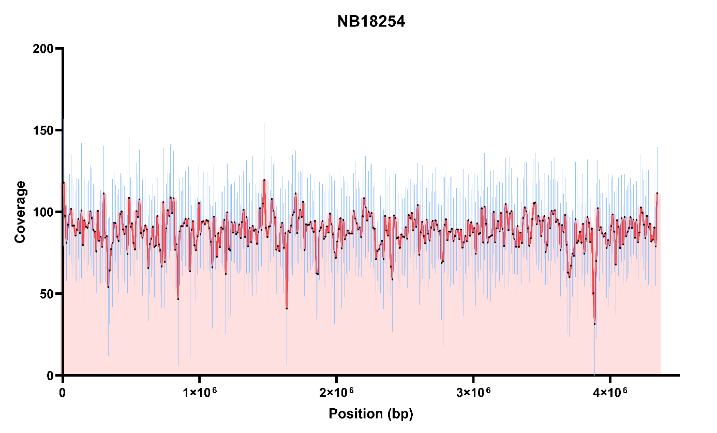

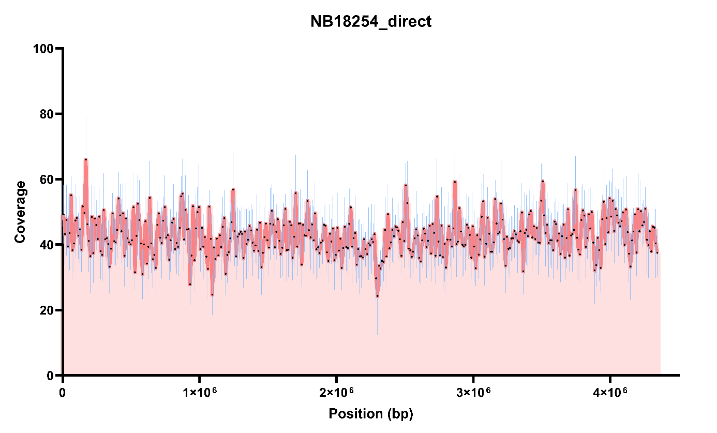

Supplement: Supplementary file 1 [file Data_Sheet_1.zip › Supplementary Material S2.DOCX]

**Mapping quality across the reference genome.**


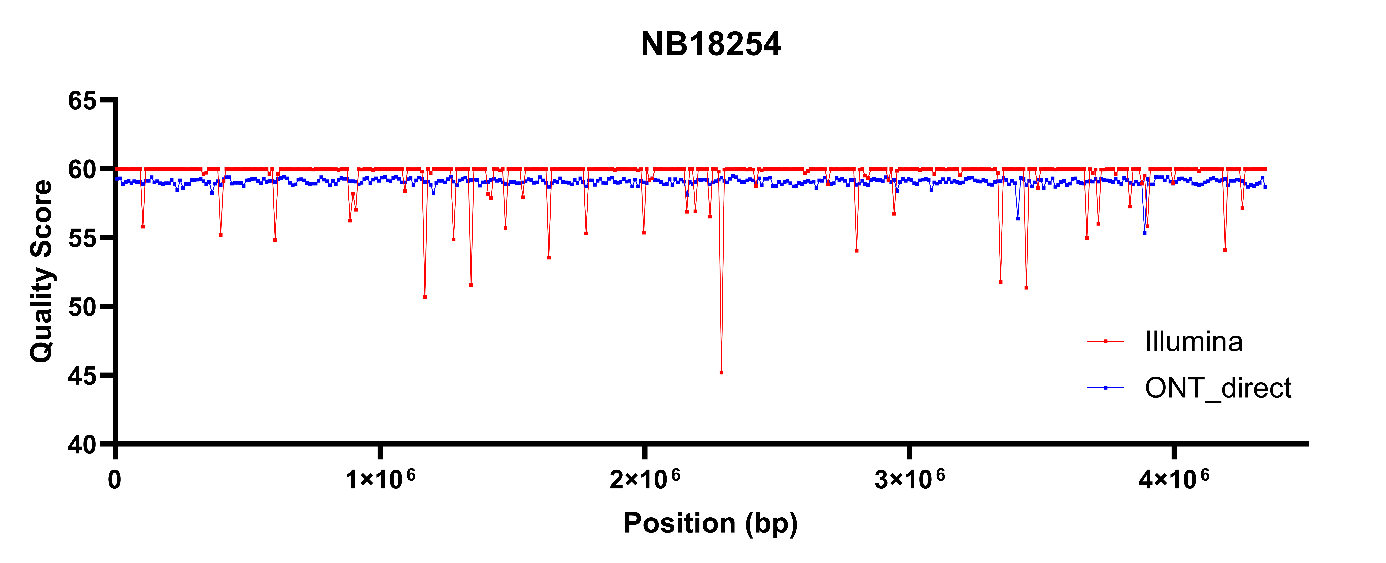

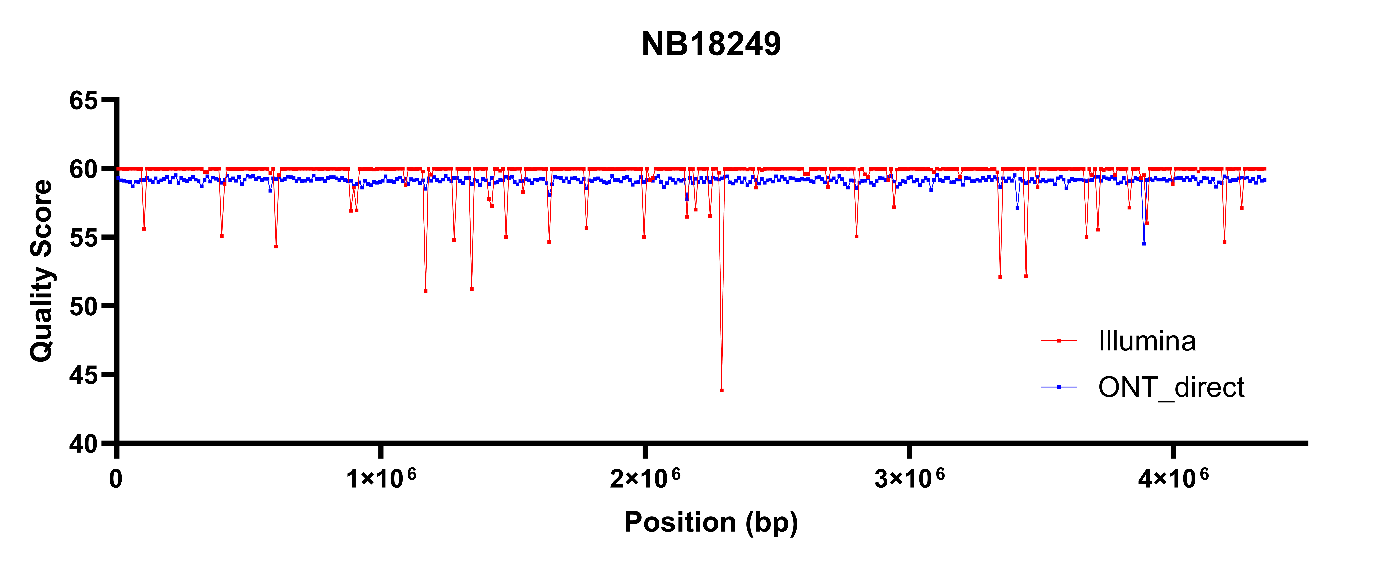

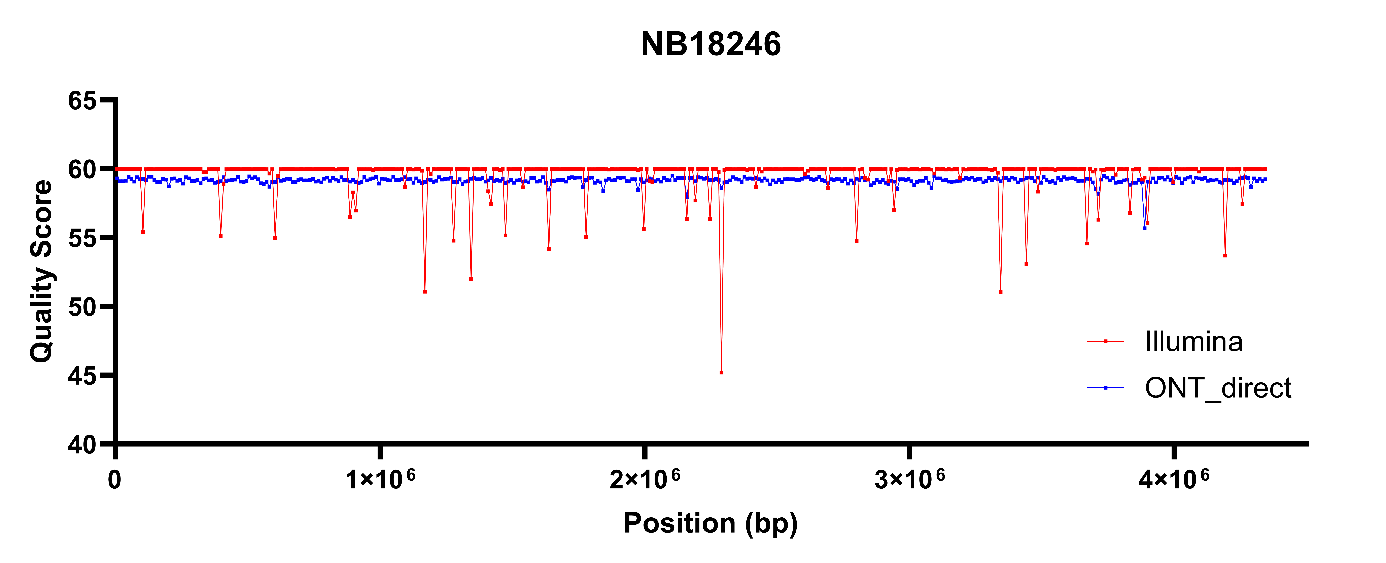

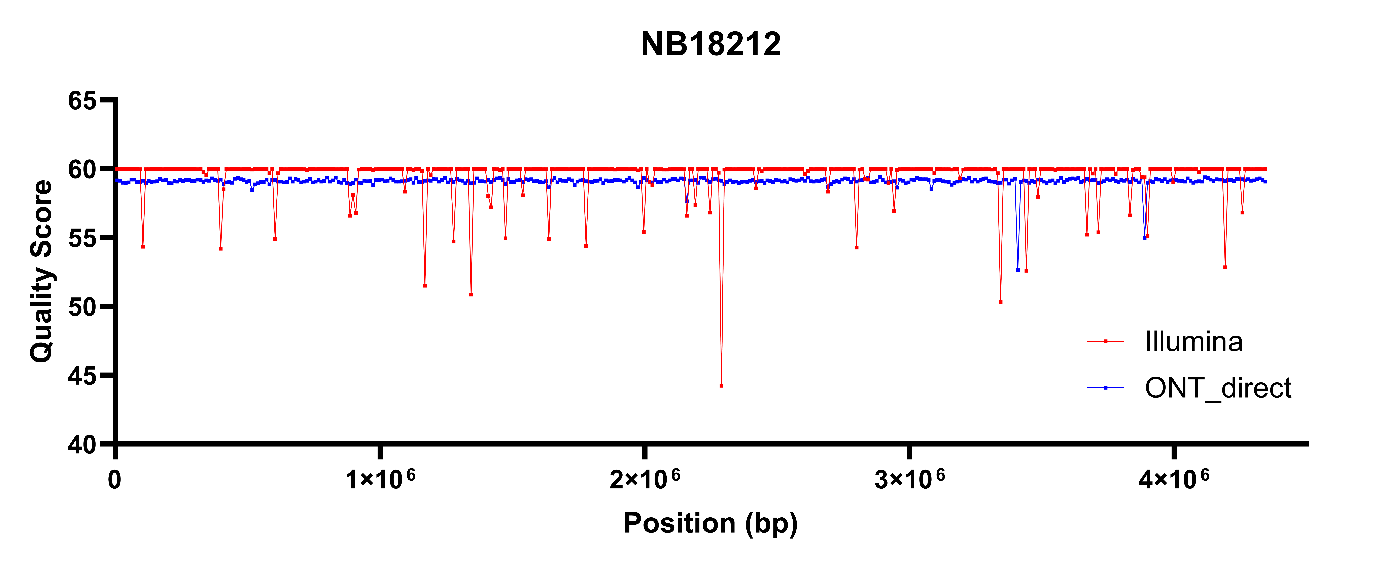

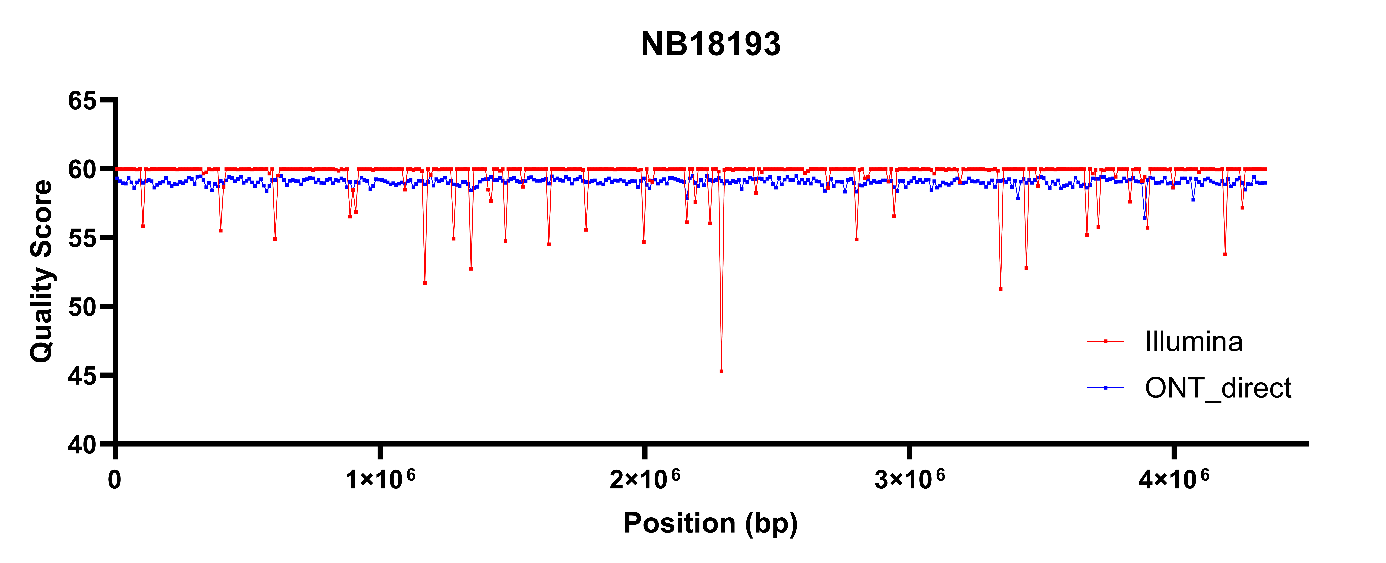

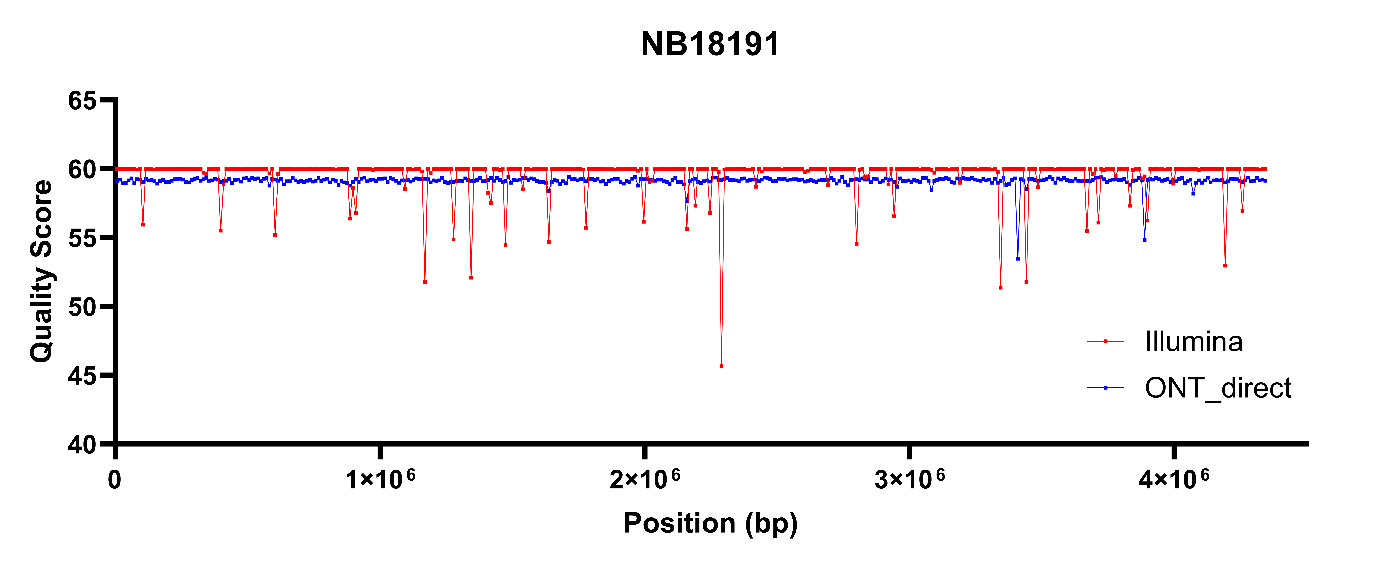

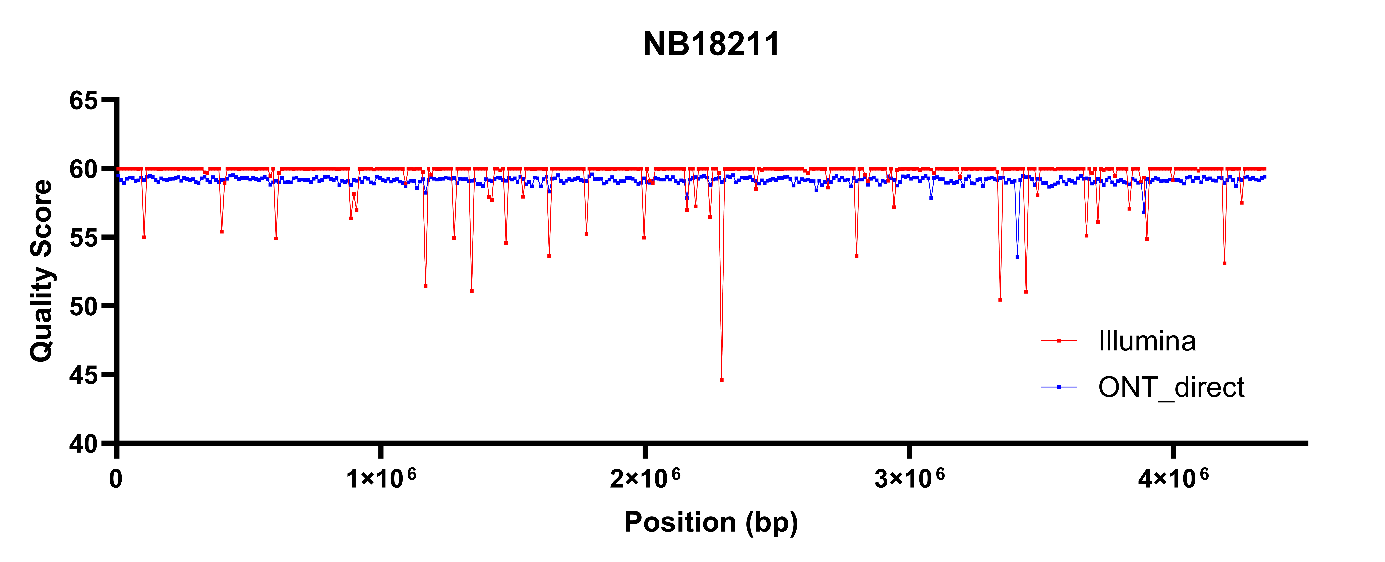

Supplement: Supplementary file 1 [file Data_Sheet_1.zip › Supplementary Material S3.DOCX]
